# Toward trustworthy healthcare AI: designing academic research for translation readiness

**DOI:** 10.1038/s44320-026-00219-4

**Published:** 2026-05-27

**Authors:** Achim Hekler, Florian Buettner

**Affiliations:** 1 Institute of Informatics, Goethe University Frankfurt, Frankfurt am Main, Germany; 2 German Cancer Consortium (DKTK), partner site Frankfurt/Mainz, a partnership between DKFZ and UCT Frankfurt-Marburg, Frankfurt am Main, Germany; 3 German Cancer Research Center (DKFZ), Heidelberg, Germany; 4 Frankfurt Cancer Institute (FCI), Frankfurt am Main, Germany; 5 Department of Medicine, Goethe University Frankfurt, Frankfurt am Main, Germany; 6 Department of Medicine II, Hematology and Oncology, Goethe University Frankfurt, University Hospital Frankfurt, Frankfurt am Main, Germany

**Keywords:** Computational Biology

## Abstract

In controlled proof-of-concept studies, healthcare artificial intelligence (AI) systems now routinely match or exceed specialist-level performance. Despite these impressive technical achievements, most systems remain confined to research settings, highlighting an opportunity to better prepare academic AI research for subsequent clinical translation. Successful translation requires attention to technical, regulatory, organizational, and infrastructural factors, ideally from the earliest stages of research. This Perspective focuses on trustworthiness as a key factor that academic researchers can address proactively. First, we outline how trustworthiness requirements differ across three stakeholder groups—patients, clinicians, and regulatory bodies—and how academic research can better align with these expectations. We then discuss key dimensions of trustworthy AI—explainability, uncertainty quantification, and evaluation—and how researchers can address them during study design. We also consider foundation models, which require adapted validation approaches. Based on this analysis, we propose a stakeholder-centered framework that emphasizes proactive integration of trust requirements during early development phases rather than post hoc considerations.

## Abstract (web summary)

In this Perspective, the author propose that academic researchers can improve translation readiness by incorporating trust requirements into early-stage study design.

## Graphical abstract

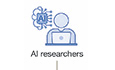

## Introduction

Healthcare AI systems now match or exceed specialist-level performance in controlled research settings, from diagnostic imaging (Esteva et al, 2017; Liu et al, 2017) to drug discovery (Jumper et al, 2021). These advances promise improved patient outcomes and solutions to challenges such as physician shortages.

Yet few systems achieve sustained clinical deployment. Although investigators continue to develop AI-enabled clinical tools at a rapid pace, few are implemented in practice, and even fewer change healthcare delivery (Balch et al, 2021). Moreover, validation standards often fall short of clinical expectations: of 521 FDA-authorized AI devices, only 4% were validated through randomized controlled trials (Chouffani El Fassi et al, 2024). This gap between technical performance in academic research and clinical readiness presents an opportunity for academic researchers to design more translation-ready studies from the outset.

Academic incentive structures contribute to this gap. The pressure for rapid publication favors studies demonstrating “AI outperforms physicians” over time-intensive multi-site validations or interdisciplinary human–AI collaboration research. This creates outputs optimized for publication impact rather than clinical translation.

**Figure 1:**
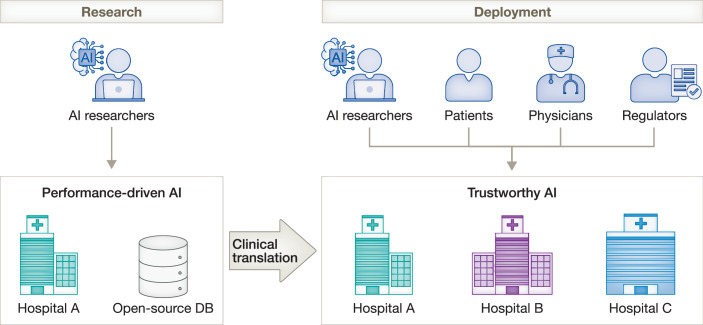
Traditional AI research focuses on optimizing technical performance metrics (such as accuracy, sensitivity, specificity, and AUROC), typically using data from single institutions or well-curated open-source databases with limited stakeholder involvement. In contrast, successful clinical deployment requires trustworthy AI systems that incorporate the trust requirements of multiple stakeholders, in particular patients, physicians, and regulators, while ensuring robust performance across diverse healthcare institutions with heterogeneous datasets.

Successful clinical translation depends on multiple factors beyond technical performance, including regulatory compliance, reimbursement pathways, data infrastructure, workflow integration, and stakeholder trust (Huang et al, 2025). Prominent examples illustrate this complexity (Table 1). For instance, Google’s diabetic retinopathy screening system achieved high diagnostic accuracy in validation (Gulshan et al, 2016), yet faced workflow integration and usability challenges in practice (Beede et al, 2020). This Perspective focuses on trustworthiness as one critical factor that academic researchers can address during study design. Trustworthiness involves dimensions such as explainability, reliability, transparency, and uncertainty quantification. Different stakeholders—patients, clinicians, and regulators—prioritize these dimensions differently (Fig. 1).

**Table 1:** Representative healthcare AI applications illustrating the research-to-practice gap.

Clinical task	Modality	Validation	Deployment status	Key barrier
Diabetic retinopathy screening (LumineticsCore) (Abràmoff et al, 2018)	Fundus images	Prospective, multi-site, FDA De Novo (2018)	Deployed in >1000 US clinical sites	Workflow adaptation required
Diabetic retinopathy screening (Google/Verily) (Beede et al, 2020)	Fundus images	Prospective validation, CE mark only	Initial deployment challenges; now scaling in LMICs	Image quality, connectivity, workflow
Prostate cancer detection (Paige Prostate) (Raciti et al, 2023)	Digital pathology	Prospective, multi-site, FDA De Novo	Routine adoption limited; mostly pilots/studies	Scanner dependency, digital infrastructure, unclear reimbursement
Skin lesion detection (Google) (Liu et al, 2020)	Dermatoscopic images	Retrospective reader studies only; no prospective clinical validation, CE mark (class 1)	CE mark only; consumer tool via Google Lens; no clinical use	Performance gaps in skin color; no FDA review; no prospective clinical validation
Sepsis prediction (Epic Sepsis Model) (Wong et al, 2021)	EHR data	Exempt as CDS software (FDA guidance); no pre-market review required	Deployed but contested	Poor external validity, alert fatigue
Oncology recommendations (IBM Watson) (Ross and Swetlitz, 2017)	Clinical notes, literature	Limited validation	Hundreds of hospitals globally; discontinued 2022	Synthetic training data; single institution bias; poor generalization to local practice

Clinical deployment status indicates whether systems have achieved sustained real-world implementation beyond research settings.

In this Perspective, we argue that academic researchers can improve translation readiness by incorporating trust requirements early in study design. First, we outline what patients, clinicians, and regulators expect from trustworthy AI (“Stakeholder-specific trustworthiness requirements”). We then discuss key technical dimensions (explainability, uncertainty quantification, and model evaluation) and what researchers should consider during study design (“State-of-the-art and implementation challenges”). We also consider foundation models, which introduce distinct validation challenges (“Foundation models in healthcare: new dimensions of trustworthiness challenges”). Based on this analysis, we propose a stakeholder-centered framework that helps academic researchers integrate trust requirements from the earliest research phases (“Recommendations for implementation-ready trustworthy AI in healthcare”). Our goal is to help academic researchers design studies that can serve as a foundation for future clinical translation.

## Stakeholder-specific trustworthiness requirements

The successful clinical translation of AI systems depends on earning the trust of diverse stakeholders, each bringing unique perspectives and requirements on trustworthiness. Understanding these priorities helps explain why technically excellent AI systems often fail in real-world deployment. Before examining stakeholder-specific requirements, we clarify key terms. Trust refers to the confidence that stakeholders place in an AI system. Trustworthiness describes properties that make a system deserving of that trust, such as reliability, transparency, and fairness. Trustworthy AI refers to systems designed and validated to possess these properties.

### Patient trustworthiness requirements: human oversight and transparent decision authority

Patient acceptance of healthcare AI follows consistent patterns across multiple large-scale studies, with human oversight as the primary determinant of trust. A Pew Research Center survey of 11,004 U.S. adults found that 60% express discomfort with healthcare providers relying on AI for diagnosis and treatment decisions (Tyson et al, 2023). This preference persists even when patients acknowledge AI’s superior diagnostic capabilities, reflecting algorithm aversion, the systematic preference for human judgment over algorithmic recommendations regardless of performance advantages (Dietvorst et al, 2015). Riedl et al demonstrated systematic decreases in trust, information disclosure, treatment adherence, and satisfaction across a spectrum from human physicians to AI-assisted physicians to standalone AI systems (Riedl et al, 2024).

Beyond human oversight, patients prioritize explainable AI systems even when transparency compromises accuracy. Benda et al found that 57.8% of participants expressed discomfort with highly accurate but unexplainable AI systems (accuracy > 98%). This discomfort increased to 76.6% for 90% accurate unexplainable systems (Benda et al, 2024). Patient transparency requirements are context-dependent and evolve with experience. Patients require adaptive explanation systems that clarify what AI predictions mean for their health outcomes and treatment options, not the computational processes behind predictions (Robinson et al, 2023). Effective transparency requires progressive approaches that provide basic information initially and unlock more detailed explanations as patients gain experience.

### Clinician requirements: evidence-based validation, clinical interpretability, and workflow integration

Physicians’ trustworthiness requirements differ fundamentally from patient needs, emphasizing rigorous clinical validation and contextual explainability that support professional decision-making responsibilities.

A 2024 American Medical Association study of nearly 1200 physicians revealed growing AI adoption, with usage increasing from 38% in 2023 to 66% in 2024 (American Medical Association, 2024). Despite this growth, more than 90% of physicians demand comprehensive validation information, including decision-making transparency, evidence of efficacy, bias management protocols, and performance validation data (Ehrenfeld, 2024). This requirement substantially exceeds current regulatory practices. An analysis of 521 FDA-authorized AI medical devices revealed that only 22 were validated using randomized controlled trials, while 144 were retrospectively and 148 were prospectively validated (Chouffani El Fassi et al, 2024). A review of randomized controlled trials on AI in clinical practice found that the predominance of single-center trials, limited reporting of demographics, and inconsistent reports of operational efficiency raise concerns about generalizability and practicality (Han et al, 2024). This systematic disconnect between regulatory approval processes and physician evidence requirements creates implementation barriers, in which technically approved systems fail to achieve clinical adoption due to insufficient validation evidence.

Physician trust also depends on how AI systems communicate their reasoning. Engineers and physicians have distinct expectations about model explainability, with clinicians emphasizing the clinical relevance of AI outputs rather than technical interpretability. Recent studies confirm that physicians were more inclined to trust and implement AI in clinical practice if they perceived the results as more explainable or comprehensible, with 70% agreeing that their level of trust increased as a result of the explanations provided (Rosenbacke et al, 2024).

However, even well-validated and explainable AI systems face adoption barriers if they disrupt clinical practice. Workflow integration constitutes a fundamental trust requirement for physicians, as they tend to reject AI systems that require duplicate data entry or disrupt established care patterns, regardless of technical performance (Giavina-Bianchi et al, 2024). The demand for seamless integration reflects physicians’ need for AI tools that enhance rather than complicate existing clinical workflows, particularly given the substantial administrative burden already imposed by current healthcare technology systems. Time constraints in clinical environments necessitate AI systems that provide sufficient information for professional evaluation while supporting rapid decision-making under cognitive load constraints (Agency for Healthcare Research and Quality, 2023).

### Regulatory requirements: validation standards and trust alignment

Regulatory frameworks establish the minimum evidence standards for market authorization, yet these requirements do not necessarily align with the trust requirements of patients and clinicians. Two contrasting regulatory philosophies shape the healthcare AI landscape: the FDA’s efficiency-oriented approach, prioritizing rapid market access, and the EU AI Act’s risk-preventive framework, emphasizing comprehensive pre-market assessment.

#### FDA pathway

Most AI/ML-enabled devices in the U.S. are cleared via 510(k) substantial equivalence (about 96.7%), emphasizing similarity to existing devices over proof of clinical utility (Joshi et al, 2024). The FDA’s January 2025 draft guidance reinforces this efficiency-focused approach through a total product lifecycle oversight framework that maintains low barriers for initial approval while strengthening post-market monitoring requirements, including uncertainty quantification and performance tracking (U.S. Food and Drug Administration, 2025). With respect to transparency and explainability, the guidance characterizes them as “important factors” rather than as mandatory requirements, using permissive language. This regulatory efficiency creates a systematic trust gap where approved systems may lack the comprehensive validation evidence that physicians and patients require for confident adoption.

#### EU AI Act

In contrast to the FDA’s efficiency-focused approach, the EU AI Act introduces more stringent validation requirements, classifying healthcare AI systems by risk levels and mandating a comprehensive pre-market assessment for high-risk applications. Most healthcare AI applications fall under this high-risk classification, including systems for patient triage, diagnostic support, and therapy recommendation. These systems require conformity assessments, CE marking, and extensive technical documentation before market placement.

Article 13 of the EU AI Act establishes detailed transparency requirements that go beyond technical specifications, mandating comprehensive “instructions for use.” These must include: system characteristics and performance limitations (communicated through uncertainty quantification), accuracy and cybersecurity metrics, known risks to health and fundamental rights, technical capabilities for explanations of outputs, performance specifications for specific patient populations, and input data requirements (European Parliament and Council, 2024).

This comprehensive approach better aligns with stakeholder trust requirements, though it introduces new compliance challenges. Successfully navigating these requirements will likely depend on building translational capacity, including interdisciplinary teams with expertise spanning early-stage AI research and regulatory pathways to market-ready products.

### Misalignment with current academic AI research priorities

Taken together, three persistent mismatches between academic AI research priorities and healthcare implementation demands inhibit translation:

#### Publication-driven research incentives

Academic incentive structures favor research that demonstrates autonomous AI performance over research on human–AI collaboration. Studies showing that ”AI outperforms physicians” generate high-impact publications and media attention, while research on AI-assisted decision-making, though more aligned with regulatory requirements and clinical reality, offers less dramatic narratives. This publication bias perpetuates a focus on standalone system development that contradicts stakeholder requirements for human oversight. Moreover, the pressure for rapid publication discourages the time-intensive process of building interdisciplinary teams with clinical, regulatory, and technical expertise, precisely the translational capacity needed to advance AI systems from early-stage research to market-ready products. This creates a self-reinforcing cycle: academic researchers optimize for publication speed and impact rather than clinical translation, perpetuating a disconnect between research outputs and implementation requirements. While industry and regulatory frameworks have shifted toward AI-based support systems with mandatory human oversight, academic research priorities have been slower to adapt.

#### Benchmark optimization and real-world generalizability

Academic AI research typically optimizes performance on curated benchmark datasets, reporting metrics such as accuracy, sensitivity, and AUROC scores. While high technical performance is necessary for clinical adoption, it is not sufficient. For example, empirical evidence on patient preferences reveals important methodological nuances and apparent contradictions. Adaptive conjoint surveys in dermatology found that patients ranked explainability (26.6% relative performance) higher than diagnostic accuracy (20.6%), with 41.6% stating they would not rely on AI systems if neither clinician nor patient could trace how decisions were made (Haggenmüller et al, 2024). Patients with prior AI experience showed even stronger preferences for explainability (29.5%) over accuracy (16.0%). However, when participants in a UK citizens’ jury study were explicitly required to choose between more accurate or more explainable systems, 86-92% preferred accuracy in healthcare contexts, while showing opposite preferences in non-healthcare domains such as criminal justice (van der Veer et al, 2021). The evidence suggests that patient preferences are highly context-dependent, and further research is needed to clarify how these preferences vary across clinical domains and patient populations. This performance-acceptance disconnect highlights the need for research reorientation toward evaluating key trustworthiness dimensions such as explainability, reliability, and uncertainty quantification, rather than optimizing isolated technical metrics.

#### Technical explainability versus stakeholder communication needs

Current AI research emphasizes computational interpretability rather than stakeholder communication needs. Technical explainable AI (XAI) research focuses on algorithmic transparency through feature attribution and model internals, yet this approach does not always satisfy stakeholder requirements. Physicians emphasize contextual plausibility over technical explanations, with studies showing that technical model interpretations do not increase physician understanding and trust (Tonekaboni et al, 2019). Patients require fundamentally different explanation approaches focused on health outcomes and treatment implications rather than algorithmic processes (Esmaeilzadeh 2020). Regulatory frameworks such as the EU AI Act demand transparency that enables appropriate human oversight, not technical documentation of computational methods. This mismatch between technical XAI research and stakeholder communication needs can create explanation systems that satisfy neither clinical utility nor regulatory requirements.

Technically capable AI systems can thus fail to achieve clinical adoption because they optimize for research metrics rather than stakeholder trust. One approach to addressing this research-to-practice gap is to reorient toward stakeholder-centered development approaches that embed trust requirements as design constraints rather than as post hoc considerations.

## State-of-the-art and implementation challenges

The stakeholder priorities in “Stakeholder-Specific Trustworthiness Requirements” reveal three technical areas central to trustworthiness: explainability, uncertainty quantification, and evaluation. Here, we examine the methodological state-of-the-art in each dimension and highlight persistent implementation challenges. While we focus on explainability, uncertainty quantification, and model evaluation as key technical implementation challenges, we acknowledge that broader ethical dimensions, including fairness, privacy, and accountability, are equally integral to trustworthy healthcare AI but exceed the scope of this Perspective.

### Explainability and interpretability

#### Methodological overview

While explainability methods have received substantial attention in the research literature, their adoption in clinically deployed systems remains limited. Most diagnostic AI systems, including autonomous diabetic retinopathy screening tools such as LumineticsCore and sepsis prediction models like Epic Sepsis Model, operate as ”black boxes” that provide binary outputs or risk scores without accompanying explanations. This disconnect between XAI research and clinical deployment underscores the implementation challenges discussed below.

Model-agnostic post hoc methods dominate healthcare XAI research. SHAP (SHapley Additive exPlanations) provides both local and global feature attributions (Lundberg and Lee, 2017), while LIME (Local Interpretable Model-agnostic Explanations) approximates complex models with interpretable local surrogates (Ribeiro et al, 2016). For medical imaging, Grad-CAM generates localization maps highlighting diagnostically relevant regions (Selvaraju et al, 2017). Intrinsically interpretable architectures offer an alternative by embedding transparency directly into model design. Classical approaches, such as decision trees, provide transparent decision rules but face limitations with high-dimensional medical data. More recent architectures, such as TabNet, employ attention mechanisms to maintain interpretability while handling complex tabular data (Arik and Pfister, 2021). This architecture enables clinicians to understand not only which features are important but also how the model combines multiple features sequentially to reach diagnostic conclusions, providing a more intuitive decision pathway that aligns with clinical reasoning processes.

An overview of commonly used XAI methods, including their mechanisms, strengths, limitations, and current state of clinical evidence, is provided in Table EV1.

#### Implementation challenges

While these methodological advances represent significant technical achievements, they primarily address technical explainability rather than the stakeholder-specific explanation needs identified in “Stakeholder-specific trustworthiness requirements”. A systematic review of XAI in clinical decision support found that while five of ten studies reported XAI increased clinician trust compared to standard AI, 3 found no significant effect, and 2 highlighted that XAI could either enhance or diminish trust depending on explanation complexity and coherence (Rosenbacke et al, 2024). This nuanced evidence challenges the assumption that more explanation inherently improves clinical adoption.

The disconnect between the developer and clinician’s mental models of XAI creates systematic implementation barriers. A longitudinal co-design study with 112 clinicians and developers identified three fundamental differences: developers prioritize model interpretability while clinicians emphasize clinical plausibility; developers treat training data as the source of truth while clinicians prioritize patient-specific context; and developers focus on discovering new patterns while clinicians rely on established medical knowledge (Bienefeld et al, 2023). These conflicting goals suggest that technically sophisticated XAI methods may fail to achieve clinical adoption, not due to implementation challenges but because of a fundamental misalignment with clinical reasoning processes. Furthermore, explanations can paradoxically degrade clinical decision-making by increasing cognitive load or reinforcing incorrect recommendations (Jabbour et al, 2023; Schemmer et al, 2023; Bernstein et al, 2023; Spitzer et al, 2022; Yu et al, 2024).

The absence of reliable ground truth for explanations introduces fundamental validation challenges that distinguish healthcare XAI evaluation from conventional domains where objective verification remains feasible. This validation deficit is particularly significant given that a comprehensive systematic review finds that 43% of healthcare XAI studies fail to assess explanation quality, while only 11% incorporate clinicians as domain experts within validation frameworks. The recently developed Clinician-Informed XAI Evaluation Checklist with Metrics (CLIX-M) represents the first systematic attempt at standardization for healthcare contexts (Brankovic et al, 2025). This 14-item checklist covers clinical, machine, and decision attributes and was developed through qualitative studies. However, this framework remains provisional and requires broader validation across diverse clinical settings and use cases.

### Uncertainty quantification

#### Methodological overview

Uncertainty quantification encompasses two fundamental categories (Hüllermeier and Waegeman, 2021; Kendall and Gal, 2017). Epistemic uncertainty reflects knowledge limitations arising from insufficient training data or inadequate model capacity, which can, in theory, be reduced through enhanced data acquisition. Aleatoric uncertainty captures the irreducible randomness inherent in the data, including biological variability and measurement noise.

Bayesian approaches provide the theoretical foundation by treating model parameters as probability distributions (Neal, 2012), but are analytically intractable. Even existing approximate inference methods, such as variational inference and Markov Chain Monte Carlo (MCMC), are computationally expensive, limiting their deployment in resource-constrained clinical environments (Blundell et al, 2015; Neal, 2011).

Given these computational limitations of Bayesian approaches, non-Bayesian methods have emerged as computationally tractable alternatives that achieve robust uncertainty estimation without requiring fundamental architectural modifications to existing deep learning systems. Deep ensemble methods generate uncertainty estimates through prediction variance across multiple independently trained models. These models use varied initializations or architectural configurations, providing effective approximations to Bayesian posterior distributions (Lakshminarayanan et al, 2017). Monte Carlo Dropout leverages stochastic regularization during inference by performing repeated forward passes with varied dropout patterns, enabling uncertainty quantification without the overhead of ensemble training (Gal and Ghahramani, 2016). Model re-calibration techniques address uncertainty miscalibration through post hoc methods such as temperature scaling and Platt scaling, correcting probability estimates to better reflect true predictive accuracy (Guo et al, 2017; Platt, 1999). Unlike ensemble approaches or Monte Carlo Dropout, post hoc calibration methods require minimal computational overhead and can be retrofitted to existing clinical AI systems, facilitating practical uncertainty quantification in deployed healthcare applications.

Conformal prediction provides distribution-free uncertainty quantification with finite-sample statistical guarantees, offering an alternative paradigm that constructs prediction sets rather than point estimates with confidence scores (Shafer and Vovk, 2008). This framework leverages conformity scores computed on calibration data to establish prediction intervals that contain the true value with specified probability coverage. However, the clinical deployment of these uncertainty methods faces fundamental challenges in stakeholder communication and in providing actionable guidance.

#### Implementation challenges

Failing to differentiate between aleatoric and epistemic uncertainty and to communicate them is a critical yet frequently overlooked barrier to the adoption of clinical AI systems. Most clinical AI systems combine both types into a single confidence score, making it unclear whether the uncertainty originates from unavoidable randomness or addressable knowledge gaps. However, different clinical actions are required for each type of uncertainty. Aleatoric uncertainty requires risk management strategies that accept biological variability, whereas epistemic uncertainty necessitates further data collection or expert consultation, or the avoidance of automated decision-making. The widespread use of heatmaps based on confidence scores intended to identify regions of medical images that require careful consideration by physicians highlights the urgent need to differentiate between the two types of uncertainty. However, these visualizations often reflect epistemic uncertainty arising from limitations in the training data or the model architecture rather than genuine clinical ambiguity. This misleads clinicians into treating the model’s knowledge gaps as medical complexity.

Furthermore, research reveals significant challenges in the effectiveness of uncertainty visualization, with studies demonstrating that clinicians often struggle to interpret uncertainty information appropriately and may ignore uncertainty-based information when making decisions (Gillmann et al, 2021; Padilla et al, 2018). These visualization failures stem from two fundamental issues: the inherently subjective nature of uncertainty interpretation and the cognitive complexity of processing uncertainty information in clinical settings. First, uncertainty representations such as confidence scores and probability ranges are interpreted differently across individual clinicians based on their experience, risk tolerance, and clinical context—a confidence score of 0.7 may be perceived as acceptably reliable by one physician but insufficiently certain by another. Second, uncertainty information requires complex cognitive processing that conflicts with the rapid decision-making demands of clinical practice, where physicians need immediately actionable guidance rather than abstract probabilistic estimates. The cognitive mechanisms underlying these failures are well-documented: uncertainty information competes for limited working memory resources under clinical time pressure (Agency for Healthcare Research and Quality, 2023).

Another fundamental challenge lies in translating computational uncertainty scores into actionable clinical guidance, as raw probabilistic estimates provide no inherent direction for appropriate clinical responses (Begoli et al, 2019). The development of uncertainty-guided clinical protocols is further complicated by context-dependent variation in acceptable uncertainty thresholds across different clinical scenarios (White et al, 2007). Emergency medicine environments may necessitate tolerance of elevated uncertainty levels due to temporal constraints and the imperative for rapid intervention, whereas elective procedural contexts typically demand substantially lower uncertainty thresholds to justify therapeutic intervention (Croskerry, 2009).

### Model evaluation

#### Methodological overview

Healthcare AI model evaluation encompasses two fundamental dimensions that together determine the success of clinical translation: systematic metric selection aligned with clinical objectives and robust performance assessment across diverse deployment scenarios.

##### Problem-aware metric selection

Current practice in healthcare AI research suffers from a fundamental misalignment where researchers routinely apply established, popular metrics (such as accuracy, sensitivity, specificity, AUROC) rather than adapting their evaluation approach to the specific characteristics and requirements of their research problem. A multidisciplinary expert consortium conducted a systematic analysis that identified three core categories of metric-related pitfalls: inappropriate problem categorization, inadequate metric selection, and flawed approaches to metric application (Reinke et al, 2024). To overcome this issue, a large international consortium of experts developed the Metrics Reloaded framework, representing a comprehensive approach to systematic metric selection in biomedical image analysis (Maier-Hein et al, 2024). This framework introduces the novel concept of a ”problem fingerprint”, a structured representation that captures all aspects relevant to metric selection, from domain interest to target structure properties, dataset characteristics, and algorithm output specifications, enabling researchers to move beyond default metric choices toward problem-specific evaluation.

##### Distribution shift and generalization challenges

Real-world robustness evaluation performance assessment across heterogeneous clinical environments, including variations in imaging equipment specifications, institutional protocols, patient demographics, and clinical workflow patterns (Balendran et al, 2025). Multi-site validation with diverse, representative datasets represents the gold standard for assessing generalization, as it reveals performance degradation patterns that remain hidden in single-center studies. Building the infrastructure and data-sharing agreements necessary for routine multi-site validation should be a priority for the field. Where access to multi-site data remains constrained, a common limitation in academic settings, synthetic corruption testing provides an intermediate approach (Hendrycks and Dietterich, 2019). While synthetic corruptions cannot replace real-world validation, they offer a pragmatic starting point for robustness assessment, preferable to single-site evaluation alone. “Recommendations for implementation-ready trustworthy AI in healthcare” discusses how stakeholder-centered development, including the early involvement of diverse clinical sites, can help address barriers to data access.

##### Evaluation of human–AI performance

Clinically meaningful evaluation requires assessing human–AI team performance rather than comparing AI systems against physicians in isolation. The relevant question for clinical practice is not whether AI outperforms physicians, but whether physicians supported by AI outperform physicians working alone, and under what conditions. This “Physician and AI” evaluation paradigm aligns with regulatory requirements for human oversight and reflects the decision-support role that AI systems occupy in clinical workflows. Study designs should therefore measure collaborative outcomes: diagnostic accuracy of the human–AI team, time-to-decision, physician confidence, and workflow integration. While traditional reader studies comparing AI versus physician performance remain common in research publications, they provide limited insight into real-world clinical utility and may perpetuate the misleading narrative that AI should replace rather than augment clinical expertise.

#### Implementation challenges

Despite methodological advances in evaluation frameworks, fundamental gaps persist between research evaluation practices and clinical implementation requirements, creating systematic barriers to successful AI deployment. While the Metrics Reloaded framework represents a significant advance in systematic, appropriate metric selection, it remains focused on technical performance measures in medical imaging. However, clinical success often depends on entirely different outcomes, including quality of life improvements, physician time savings, and cost-effectiveness measures.

Furthermore, regulatory frameworks, particularly the EU Medical Device Regulation (MDR) and EU AI Act, mandate highly specific intended use specifications that define precise user populations, clinical contexts, and inclusion criteria, directly influencing training and validation data requirements. For example, an AI-based skin cancer detection system might have an intended use specification such as: “dermatology residents photographing suspicious skin lesions with iPhone X cameras in German university hospital settings for patients referred by general practitioners.” Such specificity contrasts sharply with typical research evaluation practices that rely on retrospective datasets with broad, convenience-based inclusion criteria such as “patients with suspicious skin lesions”. Most healthcare AI research utilizes heterogeneous image collections spanning multiple institutions, imaging devices, patient populations, and clinical contexts that poorly represent the narrow specifications required for regulatory approval. This systematic mismatch between broad research validation and restrictive regulatory intended use creates implementation barriers where research-validated systems require extensive additional validation to demonstrate performance within their specified clinical context, often necessitating costly prospective studies that delay clinical deployment.

## Foundation models in healthcare: new dimensions of trustworthiness challenges

### Novel failure modes: hallucinations and emergent behaviors

Foundation models introduce novel failure modes that differ fundamentally from traditional machine learning approaches in healthcare. Unlike conventional medical AI systems, which operate within narrow, well-defined domains, foundation models exhibit emergent behaviors and generate plausible, yet potentially dangerous, medical content that resists conventional validation approaches (Moor et al, 2023).

Medical hallucination represents the most immediate and clinically dangerous manifestation of foundation model unreliability. These instances are misleading medical content that appear clinically plausible but are factually incorrect, which is particularly harmful for clinical decision-making (Pal et al, 2023). Hallucinations can be categorized as responses that contradict established medical knowledge (fact-conflicting), responses that deviate from the original query (input-conflicting), or responses that contradict the model’s own generated content (context-conflicting) (Agarwal et al, 2025). The MedHalu study, which introduced the first comprehensive medical hallucination dataset, revealed that automatically detecting medical hallucinations is very challenging: LLMs perform no better than laypeople on this task (Agarwal et al, 2025).

#### Emergent capabilities

Foundation models exhibit emergent capabilities that arise spontaneously at scale without explicit training for specific tasks, creating unprecedented challenges for healthcare AI validation. These emergent behaviors manifest as sophisticated reasoning abilities and complex problem-solving skills that exceed the models’ original training objectives, yet remain fundamentally unpredictable (Wei et al, 2022; Schaeffer et al, 2023).

Foundation models demonstrate sophisticated clinical reasoning across diverse medical specialties, including differential diagnosis generation, treatment planning, and medical literature synthesis, despite not being explicitly trained for these clinical tasks (Nori et al, 2023). The mechanisms underlying these emergent abilities remain poorly understood, making it impossible to predict when models will exhibit competent medical reasoning versus when they will generate dangerous medical misinformation.

This complicates deployment, as foundation models may possess latent abilities that exceed their validated use cases but remain undetected until triggered by specific prompt configurations (Ganguli et al, 2022). For instance, a model validated for medical literature summarization may spontaneously exhibit diagnostic capabilities when presented with patient case descriptions, generating clinically plausible yet potentially incorrect recommendations without indication that such functionality was neither intended nor validated. This creates systematic risks in which models operate beyond their validated domains while maintaining confident communication that may mislead clinicians about the reliability of their outputs.

Capabilities that emerge at certain model scales may disappear or change when models are fine-tuned or adapted for specific clinical applications, creating dynamic validation challenges that require continuous reassessment throughout the model lifecycle (Wei et al, 2022; Schaeffer et al, 2023).

#### Mitigation strategies

Addressing these novel failure modes requires a multi-layered approach combining proactive evaluation with comprehensive documentation. Red-teaming exercises, where dedicated teams systematically probe models to identify failure modes and potential harms, have emerged as a critical pre-deployment practice for foundation models (Ganguli et al, 2022). These adversarial evaluations are complemented by safety benchmarking on curated datasets designed to detect medical hallucinations and assess the reliability of clinical reasoning (Pal et al, 2023; Agarwal et al, 2025). Model auditing provides ongoing assessment of system behavior across diverse clinical scenarios, enabling detection of emergent capabilities that may arise post-deployment. Transparency documentation through model cards and AI system cards offers a standardized approach to communicate model capabilities, limitations, intended use cases, and known failure modes to clinical end-users (Mitchell et al, 2019). However, the fundamental unpredictability of emergent behaviors means that even comprehensive mitigation strategies cannot guarantee the absence of novel failure modes, underscoring the continued importance of human oversight in clinical deployment.

### Validation challenges

Unlike conventional medical AI systems, which have narrow, domain-specific applications, the broad capabilities and multi-modal nature of foundation models introduce a new level of validation complexity.

Multi-modal validation creates high barriers to evaluation, as a comprehensive assessment requires expertise across multiple medical specialties simultaneously. For example, a single multi-modal foundation model that processes dermatology images, pathology slides, patient demographics, and clinical characteristics requires validation input from dermatologists, pathologists, and potentially additional specialists for each input modality. This requirement for multiple experts makes validation exponentially more complex and resource-intensive compared to traditional single-modal medical AI systems. The coordination challenges of ensuring consistent evaluation criteria across specialties, managing inter-rater reliability between different expert groups, and integrating diverse medical perspectives create validation bottlenecks that threaten the trustworthy deployment of these systems.

Data leakage represents a widespread and challenging risk to the integrity of validation, which is particularly problematic for foundation models that are trained using extensive datasets. The scale of the training data—often consisting of hundreds of billions to trillions of tokens from diverse web sources—makes contamination detection extremely difficult, if not impossible. Medical exam questions, clinical guidelines, and biomedical literature used for evaluation may have been included in the training data, inflating performance metrics and misrepresenting the model’s trustworthiness. This creates a validation challenge in which apparent high performance may reflect memorization rather than the ability to perform high-quality medical reasoning, which could directly threaten patient safety if models are deployed based on contaminated validation results.

Agent-based LLM systems introduce validation complexity through their autonomous integration with external tools and databases (Zou and Topol, 2025). Unlike traditional AI systems, these agents dynamically select and invoke external resources. This creates non-deterministic execution pathways, where system outputs depend not only on the core LLM but also on the real-time availability and data integrity of external dependencies. Validation frameworks must therefore assess not only the agent’s decision-making algorithms but also the reliability of tool selection logic, error propagation mechanisms, and failure recovery protocols.

The difficulty of comprehensive benchmarking stems from the emergent capabilities of foundation models, which extend beyond their validated scope and generate convincing outputs in other domains. Crucially, these models offer no indication when they operate outside their validated domains or apply knowledge in unvalidated contexts. For instance, a model validated for dermatological pattern recognition may apply similar visual analysis to pathology images. This can result in seemingly expert interpretations delivered with the same confidence and clinical terminology. However, there is no indication that this functionality has been validated. Therefore, clinicians cannot distinguish between reliable, tested capabilities and potentially dangerous, unvalidated model behaviors based on output quality alone.

## Recommendations for implementation-ready trustworthy AI in healthcare

**Figure 2:**
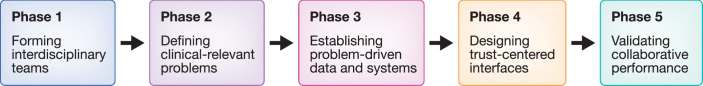
Stakeholder-centered framework for trustworthy healthcare AI implementation.

The stakeholder trust misalignments and technical implementation challenges identified in Sections "Stakeholder-Specific Trustworthiness Requirements" and "State-of-the-Art and Implementation Challenges" reveal systematic failures in current AI development approaches that prioritize technical optimization over stakeholder integration. These failures create predictable patterns: standalone systems that contradict human oversight requirements, explainability methods that increase cognitive burden rather than addressing stakeholder-specific requirements, uncertainty communication that fails to guide clinical actions, and evaluation practices that optimize for research metrics rather than clinical utility. This analysis directly informs the design of a stakeholder-centered framework that systematically addresses these fundamental disconnects (Fig. 2).

We synthesize these insights into a five-phase framework based on stakeholder-driven development. Table 2 summarizes how each implementation challenge identified in Sections "State-of-the-Art and Implementation Challenges" and "Foundation Models in Healthcare: New Dimensions of Trustworthiness Challenges" maps onto specific framework phases and proposed solutions. This framework is designed for academic AI researchers who wish to design their studies from the outset in a way that enables subsequent translation and product development. Rather, our contribution is to identify trustworthiness-specific considerations that must be embedded early in the research process, so that academic outputs can serve as a foundation for subsequent product development rather than remaining isolated research artifacts. Phase 1 addresses all implementation challenges by establishing interdisciplinary teams that embed stakeholder perspectives from the earliest stages of development. Phase 2 responds to the research-to-practice gap by prioritizing genuine clinical problems over technical classification challenges. Phase 3 addresses failures in the evaluation methodology by ensuring that data and system design align with real-world deployment contexts rather than laboratory conditions. Phase 4 directly addresses the explainability paradox and uncertainty communication issues by designing interfaces that support rather than complicate stakeholder decision-making. Finally, Phase 5 addresses the human–AI evaluation disconnect by measuring collaborative performance rather than standalone AI capabilities.

**Table 2:** Challenge-to-solution mapping linking implementation barriers (sections “State-of-the-art and implementation challenges” and “Foundation models in healthcare: new dimensions of trustworthiness challenges”) to framework recommendations (“Recommendations for implementation-ready trustworthy AI in healthcare”).

Domain	Challenge	Phase	Proposed solution
**Explainability (“Explainability and Interpretability”)**	XAI methods address technical transparency but fail to meet stakeholder-specific explanation needs	1, 4	Embed clinicians and patients in teams from inception; design stakeholder-specific explanations
	XAI increases cognitive load and may reinforce errors	4	Design trust-centered interfaces that support rather than complicate decision-making; iterative usability testing with clinical end-users
	Explanation quality rarely validated; minimal clinician involvement in XAI evaluation	1, 5	Integrate clinicians in XAI validation; validate explanation utility through human–AI team performance studies
**"Uncertainty quantification”**	Clinicians misinterpret uncertainty visualizations; cognitive processing conflicts with rapid decision-making	4, 5	Co-design uncertainty displays with clinical users; establish confidence thresholds that trigger specific clinical actions
	Context-dependent thresholds undefined (emergency vs. elective settings)	2	Define clinical context in problem specification; establish setting-specific uncertainty thresholds through interdisciplinary consensus
**"Model evaluation**”	Metric selection misaligned with clinical objectives	2	Prioritize clinically relevant endpoints (e.g., quality of life, time-to-diagnosis, cost-effectiveness)
	Distribution shift causes performance degradation outside the development environment	3, 5	Prospective data collection reflecting deployment contexts; multi-site validation as gold standard
	Human-AI team dynamics not been evaluated	5	Validate collaborative performance (clinician + AI) rather than standalone AI; measure workflow integration, cognitive load impact, productivity changes
	Research validation mismatches the regulatory intended use specifications	2, 3	Develop comprehensive intended use specifications early
**Foundation models (“Foundation models in healthcare: new dimensions of trustworthiness challenges”)**	Medical hallucinations generate plausible but factually incorrect content	1, 5	Include domain experts for output verification; implement hallucination detection protocols; and continuous monitoring in deployment
	Emergent capabilities unpredictable; models may operate beyond the validated scope	2, 5	Define precise functional boundaries in intended use; implement guardrails preventing out-of-scope operation; continuous validation throughout the lifecycle
	Multi-modal validation requires coordination across multiple specialties	1	Form interdisciplinary teams with expertise spanning all input modalities

These five phases are:

### Phase 1: forming interdisciplinary teams

The foundation for stakeholder-centered AI development requires establishing interdisciplinary teams before technical development begins. Minimum team composition includes a technical lead with healthcare AI expertise and a practicing clinician to ensure clinical relevance and workflow integration. Full teams expand to include patient representatives, implementation specialists, and additional clinical practitioners from target specialties to address diverse stakeholder needs and workflow variations.

Realizing this vision requires institutional infrastructure that facilitates sustained collaboration across disciplines. Standing networks connecting clinical institutions with AI research groups can provide the secure data environments and governance structures necessary for meaningful interdisciplinary work. Such networks enable physicians to contribute clinical expertise and access to AI-ready data, while data scientists and model developers gain exposure to genuine clinical problems rather than relying on convenience datasets. Successful examples from other industries demonstrate that multi-site collaboration within appropriate regulatory frameworks is achievable; the challenge lies in building comparable infrastructure for healthcare AI. Funding agencies and institutions can accelerate this process by incentivizing collaborative, translation-oriented research over single-center algorithm development.

### Phase 2: defining clinically relevant problems

Rather than pursuing technical classification tasks, teams identify genuine clinical decision problems through collaborative analysis of clinical workflows. This process examines where practitioners face diagnostic uncertainty, time pressure, or limited expertise that create workflow bottlenecks or compromise patient outcomes. The team develops comprehensive intended-use specifications that define precise functional boundaries, operational parameters, and clinically relevant endpoints, prioritizing patient outcomes over algorithmic performance metrics.

### Phase 3: establishing problem-driven data & systems

Clinical partners guide problem-targeted data collection, providing access to relevant patient cohorts and ensuring that the data accurately reflect real-world decision-making scenarios. Prospective data collection captures genuine clinical context, while retrospective approaches remain viable when they accurately represent target scenarios. High-quality data standards prioritize verification over volume, with clinical expertise validating labels against established gold standards. System development proceeds through close collaboration between technical and clinical team members, ensuring design decisions align with workflow requirements.

### Phase 4: designing trust-centered interfaces

Post hoc explanation methods generate stakeholder-specific interpretations: patient explanations provide comprehensible summaries in accessible language, while clinician explanations apply feature attribution techniques with confidence scores for professional evaluation. Uncertainty quantification methods establish confidence levels that trigger specific clinical actions, including additional testing, specialist referral, or treatment recommendations. Decision threshold optimization can be evaluated through retrospective case studies with simulated clinical scenarios.

### Phase 5: validating human–AI team performance

Validation examines collaborative dynamics between AI systems and clinical practitioners rather than isolated algorithmic performance. Human–AI team performance assessment evaluates the quality of clinical decision-making with AI support compared to traditional practice, measuring improvements in patient outcomes and workflow integration through time-to-diagnosis, cognitive load impact, and productivity changes, in addition to technical metrics. Robustness validation assesses performance across diverse clinical environments, with multi-site validation representing the gold standard, though pilot studies can simulate robustness challenges through artificial perturbations when multi-site data collection is cost-prohibitive.

### Iterations and feedback loops

While the five phases are presented sequentially, real-world development involves iterations between phases. However, not all iterations are appropriate. A common anti-pattern in academic AI research is adapting the problem definition to fit available data. This approach undermines translation from the outset by reversing the logic of stakeholder-centered development. The intended use specification (Phase 2) should be defined collaboratively with clinical experts before data collection begins and should remain stable throughout development. Legitimate iterations, by contrast, arise when later phases reveal genuine issues that require revisiting earlier decisions. Usability testing (Phase 4) may uncover workflow integration problems that necessitate refining the interface or even reconsidering specific functional requirements. Validation studies (Phase 5) may identify performance gaps in particular patient subgroups, requiring additional targeted data collection (Phase 3). Such feedback loops are expected and necessary—they represent learning from stakeholder engagement rather than post hoc rationalization of data-driven decisions.

### Case study

To illustrate how these framework phases translate into practice, we consider the case study of LumineticsCore.

LumineticsCore (Digital Diagnostics) became the first FDA-authorized autonomous AI diagnostic system in 2018, achieving deployment across >1000 U.S. primary care sites. Its trajectory illustrates several framework principles:

**Phase 1 (teams):** Led by a physician-scientist; ethical framework developed with bioethicists before regulatory submission.

**Phase 2 (problem):** Addressed genuine clinical need (diabetic retinopathy screening where ophthalmologists are unavailable) with precise intended use specification.

**Phase 3 (data):** Prospective trial at 10 diverse primary care sites with representative demographics and prognostic reference standard.

**Phase 4 (interface):** Binary output for non-specialists; workflow integration explicitly considered.

**Phase 5 (validation):** Subgroup analysis and post-deployment monitoring documented.

**Contrast:** The Epic Sepsis Model, deployed without target-environment validation, missed 67% of cases with high alert burden, illustrating the consequences of insufficient attention to deployment context.

## Conclusions

Healthcare AI systems consistently achieve specialist-level performance in controlled research environments, yet the overwhelming majority fail to achieve sustained clinical deployment. This perspective examined trustworthiness as a critical dimension of the research-to-practice gap, investigated stakeholder requirements, and revealed misalignments with current AI research priorities. We further discussed technical implementation challenges across key trust dimensions (explainability, uncertainty quantification, and model evaluation) and examined novel challenges introduced by foundation models.

Based on this analysis, we propose a stakeholder-centered framework that emphasizes the proactive integration of trust requirements in early development phases rather than post hoc considerations. While trustworthiness alone does not guarantee successful translation (successful clinical deployment also requires addressing scalability, regulatory compliance as a medical device, product lifecycle management, and rigorous data quality control), this five-phase approach provides a necessary foundation for clinical adoption by reorienting fundamental development from technology-driven to stakeholder-driven.

## Supplementary information

Peer Review File

Table EV1
