# Supplementary material for: Toward trustworthy healthcare AI: designing academic research for translation readiness
Source: Mol Syst Biol. 2026 May 27;22(8):1201–13. doi: 10.1038/s44320-026-00219-4 (PMC13434723; doi:10.1038/s44320-026-00219-4)
Supplement: Supplementary file 1 [file 44320_2026_219_MOESM1_ESM.pdf]

molecular systems biology

Toward Trustworthy Healthcare AI: Designing Academic Research for Translation Readiness

Achim Hekler and Florian Buettner

Corresponding author(s): Florian Buettner (florian.buettner@dkfz-heidelberg.de) , Achim Hekler (achim.hekler@dkfz.de)

Review Timeline:

Submission Date:	10th Sep 25
Editorial Decision:	31st Oct 25
Revision Received:	4th Feb 26
Editorial Decision:	5th Apr 26
Revision Received:	20th Apr 26
Accepted:	24th Apr 26

Editor: Jingyi Hou

Transaction Report:

(Note: With the exception of the correction of typographical or spelling errors that could be a source of ambiguity, letters and reports are not edited. Depending on transfer agreements, referee reports obtained elsewhere may or may not be included in this compilation. Referee reports are anonymous unless the Referee chooses to sign their reports.)

31st Oct 2025

Manuscript Number: MSB-2025-13347
Title: Trustworthy AI in Healthcare: A Comprehensive Analysis of the Research-to-Practice Gap
Author: Achim Hekler
Florian Buettner

Dear Florian,

Thank you for taking the time to write this interesting article for Molecular Systems Biology. We have now received reports from three reviewers who agreed to evaluate your manuscript.

Overall, the reviewers found your manuscript to be relevant and interesting. However, they also raised several points and suggestions that could further strengthen the paper.

Without reiterating all the comments in detail, some of the key points include:

- Reviewers #1 and #3 recommended incorporating more concrete examples and improving the operationalization of the proposed five-phase framework.

- Reviewer #2 noted that the current version may have downplayed or overlooked factors beyond trustworthiness that are also critical for successful AI implementation. In response to these comments, we recommend that you acknowledge these additional factors in your revision and moderate claims that might give readers the impression that trustworthiness alone bridges the research-to-practice gap.

- In line with Reviewer #2's feedback, we recommend changing the article type from a "Review" to a "Perspective."

We encourage you to carefully consider the reviewers' additional comments during your revision, while leaving it to your discretion whether to incorporate specific suggestions.

On a more editorial level:

1. Please submit the manuscript in .docx format. Remove all figures from the manuscript file and upload each as a separate, high-resolution image file. Our graphic designer will redraw the figures based on these.

2. Provide up to five keywords in the manuscript file.

3. Include a "Disclosure and Competing Interests Statement." Any employment in a biotechnology company must be explicitly stated in this section.

4. Add the missing callout for Figure 2.

5. The references need to be formatted according to the Molecular Systems Biology reference style. Please list up to 10 co-authors of a paper before adding et al. in the reference list. Citations should be listed in alphabetical order.

6. Add funding information in the Acknowledgements section, ensuring it is consistent with the details entered in the online submission system.

7. Section order should be arranged in the following order: Title page - Abstract -Keywords - Introduction - Acknowledgements - Disclosure and Competing Interests Statement - References -Figure Legends - Table(s)

When submitting a revised version of your manuscript, please attach a covering letter giving details of the way in which you have handled each of the points raised by the referees. A revised manuscript will be once again subject to review.

We look forward to receiving your revised manuscript soon.

Kind regards,
Jingyi

Jingyi Hou, PhD

Senior Editor
Molecular Systems Biology

We realize that it is difficult to revise to a specific deadline. In the interest of protecting the conceptual advance provided by the work, we recommend a revision within 3 months (29th Jan 2026). Please discuss the revision progress ahead of this time with the editor if you require more time to complete the revisions.

As a matter of course, please make sure that you have correctly followed the instructions for authors as given on the submission website.

*** PLEASE NOTE *** As part of the EMBO Press transparent editorial process initiative (see our Editorial at https://dx.doi.org/10.1038/msb.2010.72), Molecular Systems Biology publishes online a Review Process File with each accepted manuscripts. This file will be published in conjunction with your paper and will include the anonymous referee reports, your point-by-point response and all pertinent correspondence relating to the manuscript. If you do NOT want this File to be published, please inform the editorial office at contact@molsystbiol.org within 14 days upon receipt of the present letter.

Reviewer #1:

Summary of the Manuscript
This review manuscript provides a timely and comprehensive overview of the critical barriers that impede the translation of artificial intelligence (AI) from research environments into routine clinical practice. The authors address the persistent research-to-practice gap by discussing the perspectives of three key stakeholder groups, including patients, clinicians, and regulatory bodies, and framing "trustworthiness" as a central requirement for successful implementation. The manuscript convincingly argues that explainability, interpretability, and reliability are as important as, if not more important than, raw predictive accuracy for AI systems intended for healthcare applications.
The review also highlights the disconnect between the generalizable nature of AI research models and the highly specific, context-dependent requirements of clinical and regulatory environments. Finally, the authors propose a stakeholder-centered framework for developing AI models that are trustworthy, transparent, and implementation-ready. The topic is both novel and highly relevant, addressing one of the most pressing challenges in the field of digital health. However, the manuscript would benefit from additional analytical depth, improved structure, and greater use of concrete examples to strengthen its overall contribution.

Major Comments
1. Need for a Comprehensive Overview Table
While the text includes numerous illustrative examples, they are dispersed throughout and challenging to track. A summary table consolidating these examples would significantly enhance the reader's understanding of the current landscape of AI in healthcare. The table could include the following columns: clinical task/question, data modality, AI algorithm or architecture, dataset size, validation strategy (internal/external), clinical application context, advantages, and limitations. This will help readers quickly grasp the diversity and maturity of existing applications.

2. Lack of Concrete, Traceable Case Studies
Several sections, particularly Section 3.1 ("Methodology Overview"), discuss general AI methods such as decision trees and random forests but do not cite specific real-world implementations. Adding traceable examples, including references to published studies or deployed systems, would anchor the discussion and help illustrate the practical challenges these methods face in clinical translation. Consider highlighting a few high-impact use cases (e.g., radiology, pathology, dermatology, or cardiology) where AI has either succeeded or failed in real-world adoption.

3. Challenge-to-Solution Mapping
Section 5 ("Recommendations for Implementation-Ready Trustworthy AI in Healthcare") offers valuable conceptual guidance, but it lacks direct linkage to the specific barriers identified in Sections 3 and 4. To enhance the paper's practical utility, include a challenge-to-solution mapping table or a straightforward narrative linking each identified challenge (e.g., data heterogeneity, lack of explainability, regulatory uncertainty) to the corresponding recommendation or proposed mitigation strategy.

4. Figures and Tables Require Strengthening
The visual components are currently underdeveloped. Figures could be made more informative and integrative, summarizing

frameworks, data flow, or stakeholder relationships. Tables summarizing XAI methods, regulatory frameworks, or implementation barriers would enhance accessibility and help readers synthesize the dense narrative content.

5. Operationalization of the Proposed Framework
The proposed five-phase stakeholder-centered framework is conceptually well-structured but remains theoretical. The manuscript would benefit from the inclusion of at least one detailed case study or pilot example. For instance, the clinical development of an AI diagnostic tool in radiology or pathology to demonstrate how this framework can be applied in real-world settings, from model design through to regulatory approval and clinical integration.

6. Need for Critical Evaluation of Evidence
Although the manuscript cites extensive secondary literature, much of the discussion remains descriptive. Assertions such as "patients consistently demonstrate willingness to sacrifice accuracy for explainability" should be supported by empirical evidence, ideally surveys or behavioral studies, with commentary on sample diversity, study design, and contextual factors. A more critical appraisal of existing data would substantially increase the review's analytical rigor and credibility.

7. Regulatory Comparison Lacks Depth
The comparison between the FDA and the EU AI Act is insightful but somewhat superficial. The discussion should be expanded to examine how the regulatory philosophies, efficiency-oriented (FDA) versus risk-preventive (EU), affect innovation ecosystems, clinical adoption speed, and patient safety culture. Including a comparative table summarizing these contrasts and their implications for AI development and approval would make this section far more impactful.

8. Synthesis of Explainable AI (XAI) Techniques
Section 3.1 lists several explainability techniques (e.g., SHAP, LIME, Grad-CAM) but does not integrate them into a comparative framework. Adding a summary table comparing key XAI methods, including interpretable models such as TabNet, based on criteria such as type (post-hoc vs. intrinsic), interpretability level, computational cost, limitations, and clinical applicability, would provide clarity and practical insight.

9. Insufficient Discussion on Mitigation of Emergent Behaviors
The section on hallucinations and emergent behaviors of foundation models correctly identifies these as critical risks but lacks discussion of mitigation strategies. The authors should expand this by referencing current best practices such as red-teaming exercises, model auditing, safety benchmarking, and the use of AI system cards for documentation. Positioning the proposed framework in relation to these tools would clarify how it complements or extends existing approaches.

10. Broader Ethical Considerations
While the paper's focus on "trustworthiness" is commendable, it underrepresents related ethical dimensions such as fairness, privacy, accountability, and transparency-all integral to stakeholder trust. These aspects should be explicitly discussed, particularly regarding how regulatory frameworks operationalize them and how they influence clinical acceptance and patient equity.

Minor Comments
1. Readability and Sentence Structure
Some sentences are overly long and concept-heavy, affecting readability. For instance, "...with discomfort increasing to 76.6% for 90% accurate unexplainable systems [17]" could be simplified or divided for clarity. A careful editorial pass focusing on sentence flow and paragraph structure would improve accessibility.

2. Clarification of References and Terminology
Certain references are insufficiently contextualized. For example, "Article 13 establishes detailed transparency requirements" should specify that this refers to Article 13 of the EU Artificial Intelligence Act, with a concise summary of its main provisions. Similarly, define abbreviations such as "technical XAI" upon first mention.

3. Citation Errors
There appears to be a citation error around the MedHalu dataset. Please verify and correct the reference.

4. Overly Detailed Methodological Sections
Some methodological explanations, particularly in Section 3, could be shortened or moved to supplementary material to improve the flow and focus of the main text.

5. Clarify Novel Contributions
The Conclusion should explicitly state how this review differs from prior work on trustworthy AI in healthcare (e.g., through its stakeholder-centered perspective or integration of foundation model analysis).

6. Figure Citation
Figure 2 is referenced in the captions but omitted in the main text. Please ensure that it is cited and discussed at the appropriate point.

7. Missing Reference
In Section 4.1 ("Medical Hallucination"), the final sentence lacks a reference. Please verify and add the appropriate citation.

8. Sentence Length and Readability
Several paragraphs contain lengthy, complex sentences. Breaking them into shorter, more concise statements will improve comprehension and maintain reader engagement.

Overall Assessment
This is a well-conceived and potentially high-impact review addressing one of the most critical bottlenecks in healthcare AI adoption-the trust and translation gap. The manuscript's strength lies in its conceptual framing and comprehensive stakeholder perspective. However, its impact and utility would be significantly enhanced by more structure, concrete examples, synthesis tables, and critical engagement with empirical evidence. With these revisions, the manuscript could become a seminal reference on trustworthy AI in clinical practice.

Reviewer #2:

In the manuscript entitled "Trustworthy AI in Healthcare: A Comprehensive Analysis of the Research-to-Practice Gap" the authors provide an article that partially is a review and partially a perspective or commentary.

The topic, the research-to-practice gap is indeed an important topic in medical AI. The authors put trustworthy AI into the focus as the major solution to close the gap. While trustworthiness certainly is required for medical AI applications in real-world applications, the authors dismiss other important topics required to close the gap. Further, the section about foundation models seems somewhat disconnected from the rest of the manuscript.
Following are points to be considered:

Two major aspects are missing:
scalability and generalizability.

Also there is debate, whether reliability, transparency, fairness and integration are truly part of trustworthiness or whether they are all standing for themselves as aspects that need to be addressed in addition to trustworthiness. E.g. an algorithm can be trustworthy in a limited domain, but not necessarily generally fair. Similarly, integration is not always reached, yet an AI can be trustworthy in a niche application.

General Comments:
In the reviewer's view, there are major things mixed or misaligned. Trustworthiness is a basic requirement for any successful application of health care AI. In the past, this was certainly not always the case, but it is clear as of now. As a consequence, it should not be dismissed anymore. Certainly, if missing, trustworthiness can be one reason for AI not being successfully integrated into real-world applications. Yet, it is by far not the only one and often not the most important reason for lack of implementation and part of the gap.
There are important other guardrails for AI to be implemented on the way towards clinical application that are not well addressed in this manuscript about the gap between research projects (often far too small in scope) and real-world applications that have nothing to do with trustworthiness. As written, this review implies that missing trustworthiness explains the gap and fixing solely trustworthiness will overcome this problem.
Other major topics that would have to be addressed, when talking about the gap:
1. The guardrail system of the European AI act has many aspects that are required to close the gap, often seen by academic researchers as imposed hurdles, while in reality most AI modelers just don't understand that AI in medicine cannot be based on "playing around with cool algorithms".
2. The regulations of AI as a medical device. Another area often dismissed by academic AI experts.
3. The lifecycle issue of AI as a health care product, which is another area not well or not at all understood by many academic researchers in the AI model and algorithm space.
4. The lack of understanding of the enormous requirements for data QC in the health care sector, which is dismissed - this reviewer would estimate - by more than 75% of AI modelers in the biomedical AI field.
Either, the authors choose to address more comprehensively the reasons for the gap or, alternatively, the authors could choose to focus more on the topic trustworthiness and its role in developing AI for medicine and health care. But focusing only on trustworthiness might be seen somewhat as a limitation, as trustworthiness has to be seen as a prerequisite for any future medical AI application and there is quite some literature about this topic already.

Specific comments to the text sections:

Paragraph 2.1.
While certainly interesting, this topic does only indirectly speak to the gap itself.

AI-based medical devices developed as support systems for physician decision making circumvent the issue addressed here already. Mainly due to the fact that experts can cope better with grey box applications, while white box applications are often not performing well. Again, Blackbox applications are not an option for clinical application, if one follows the guardrails as they are outlined in e.g., the European AI act or the regulations for AI-based medical devices since explainable AI is a prerequisite for real-world application.

Paragraph 2.2.
Again, while the context is really interesting to read, it does not address the gap directly.
In this context, it is also interesting to note that the FDA is aware of the fact that previous authorizations of AI-based medical devices need to be reevaluated since they do not fulfill today's guardrails for AI-based medical support systems anymore. As such, this paragraph addresses mainly the mistakes of the past.
The last paragraph of section 2.2 illustrates the reasons for failure. Yet again, this experience has been utilized in updated requirements in the aforementioned guardrails (which are seen as insurmountable obstacles by academic AI experts, mainly because dealing with these issues is not very appealing and reduces the flow towards high-impact publications).

Paragraph 2.3.
The last sentence in the section entitles EU AI Act illustrates a little bit the narrative of this review. Of course, this sentence is one explanation, why things are slower, if you have to develop systems that close the gap, while we all actually do not know this yet, since there are no AI-based medical devices put on the market following these new guardrails in Europe.
Another way to look at this could be that we need more experts that can address the tasks necessary to elevate an AI-based product from TRL1-3 to TRL7-9. So, the gap is more in how do we really develop the path towards real products. This is less an academic issue, but a translation issue with too few players and too few experts currently available.
Other industries are solving similar tasks and they find or even already found solutions.
This reviewer would respectfully disagree with the last sentence in the paragraph "Regulatory Frameworks Versus Physician and Patient Trust Requirements" In my view the European AI Act actually addresses this topic.

Section 2.4. First Paragraph.
This reviewer respectfully disagrees. While there has been an enormous push for autonomous AI products in the past, with the advent of regulations requiring explainable trustworthy AI, this has already shifted towards AI-based products with human oversight AI-based medical support systems.
This reviewer would again respectfully disagree with the paragraph entitled "Technical Approval Versus Clinical Acceptance" This paragraph implies to play technical against clinical acceptance values. This is actually not what the guardrails suggest. Both need to be addressed equally. An AI-based test that has low accuracy, even if explainable, will not survive the product development process. However, what seems more important is to address both generalizability and scalability to be included into the development process. Both require that technical and clinical acceptance values are sufficiently high to better estimate real world application.
The paragraph entitled "Technical Explainability versus Stakeholder Communication Needs" makes one important statement: "Regulatory frameworks such as the EU AI Act ....". Again, while the provided information is interesting, it distracts from the potential these new guardrails provide to guide AI-development away from the "academic play ground" to real product developments. In this context, it might be more important to mention that purely academically-driven research in this area will likely keep the gap as it is not addressing the next necessary steps, but often fighting them.

Section 3:
The statements in the first sentence of paragraph 3 are debatable. Again, trustworthiness is certainly one major aspect, but not the only one. Focusing the discussion in this manuscript only in trustworthiness, with an unexplained introduction of three areas that are addressed (explainability uncertainty quantification, and evaluation) leaves out that other aspects also need to be addressed to close the gap. This reviewer is not convinced that the gap will be closed, even if these points are all addressed.

Section 3.3
In the paragraph entitled "Distribution Shift and Generalization Challenges" this reviewer again thinks that the direction is not entirely helpful. The last sentence implies that multi-site validation remains resource-prohibitive, while generalization of AI-based approaches actually requires exactly this. Rather than spending time on synthetic corruption testing, wouldn't it be better to develop the necessary development environment that really address distribution shift and generalization challenges? This reviewer thinks that the manuscript is not sufficiently pro-active into the real-solution aspects.
This is similarly true for the paragraph Evaluation of Human-AI-Performance. Yes, the direct comparison is not leading anywhere, but it would be important to better describe what is really required: Physician +/- AI-based support systems, rather than criticizing the past.

In the paragraph "implementation challenges":
Again, this reviewer thinks that the authors missed out on opportunities to draw a picture of the future.
As stated earlier by this reviewer, the real problem is that academic AI modeler are often not interested in bringing their work to biomedicine, but rather cranking papers focusing on one algorithm after the other on medically often meaningless - often open access - datasets. This focus on paper rather than impact creation is one main reason for the gap. As long as the sentence "I

don't want to deal with physicians, I just need some data to try my best algorithm" is a standard in the field of academic AI research, the gap will not close.
If at all, one needs to sketch roadmaps, how those experts that handle the medical tasks (first of all physicians) enter into meaningful discussions with data scientists to generate AI-ready data, followed by data analysts to provide QC-ed data to model experts that develop AI based on best model + best data. This only is provided in section 5 without a lot of detail of the "how to".
We need standing networks across institutions that address these issues in secured environments where experts can work together despite existing regulations that might be experienced as hurdles, instead of trying to address them in single center AI-studies.

Paragraph 4
The first paragraph under 4.1. nicely summarizes the enormous difference between foundation models and conventional AI. This reviewer would strongly suggest to focus on conventional AI. The well-made points of this short two sentences question trustworthiness in foundation models significantly. Sometimes less is more.

Paragraph 5:
While this reviewer actually likes the idea to provide a framework for development, the proposed structure misses out on important aspects.
* Link to TRL system
* Link to regulatory frameworks and guardrails
* Link to development paths for products (AI-based medical support systems)
* Aspects of product life cycle, different kinds of shifts (distribution shifts, etc.)

While the text for the five phases is easy to understand, it stays often rather vague. E.g., for validation, there are already clearly defined regulations and pathways towards product development that need to be fulfilled and this would need some more description of the components. There is no doubt that phase 5 validation is of utmost importance, but it remains unclear from the text in this manuscript how this would look like in a project that would close the gap.

Reviewer #3:

Summary

This manuscript provides a comprehensive review of the persistent gap between research achievements and clinical deployment of artificial intelligence (AI) systems in healthcare. The authors argue that while AI systems frequently reach or exceed specialist-level performance under controlled research conditions, the majority fail to achieve sustained real-world implementation. The review attributes this "research-to-practice gap" primarily to deficits in trustworthiness, which is examined through the lens of three key stakeholder groups - patients, clinicians, and regulators.

The authors synthesize evidence across explainability, uncertainty quantification, and model evaluation, identifying common methodological and communication gaps that undermine stakeholder trust. The paper further discusses foundation models as a new class of challenges due to emergent behaviors, hallucinations, and complex validation requirements.

Methodologically, the review adopts a narrative synthesis of recent literature, regulatory documents, and benchmark studies, integrating quantitative data from surveys, validation studies, and systematic reviews. The proposed concept is a stakeholder-centered five-phase framework for trustworthy healthcare AI development encompassing interdisciplinary collaboration, clinically relevant problem definition, data/system alignment, trust-centered interface design, and human-AI team performance validation.

General Remarks

The manuscript is well-written, logically structured, and highly relevant to current discussions on AI implementation in healthcare. It effectively bridges technical and stakeholder perspectives, making it valuable for interdisciplinary readers in medicine, computer science, and health policy.

The key conclusions are convincing overall and supported by appropriate evidence. The review offers a conceptual and

methodological advance, reframing trustworthy AI as a stakeholder-centered design problem rather than a post-hoc validation issue. The inclusion of foundation models and their emergent challenges adds timely depth.

In terms of novelty, while many prior reviews have discussed AI ethics and explainability, this manuscript's integration of stakeholder trust requirements with technical implementation barriers represents a significant conceptual contribution. The proposed five-phase stakeholder-centered framework offers practical guidance and may influence future interdisciplinary development practices.

This work will interest audiences in: Biomedical AI research and translation; Regulatory science and digital health policy; Clinical implementation and digital medicine; AI ethics and human-AI interaction research, and beyond...

Major Points

1. Framework Validation

The proposed five-phase framework is conceptually sound, but it remains theoretical. The authors could strengthen their argument by referencing any empirical or case-study evidence where similar stakeholder-driven frameworks have improved AI deployment (e.g., clinical AI co-design studies). Furthermore, possible feedbacks/feedforwards/iterations of the proposed five phases should be discussed.

2. Depth of Discussion on Foundation Models

The section on foundation models identifies key risks (hallucinations, emergent behaviors, data leakage) but could be strengthened by suggesting practical mitigation strategies (e.g., benchmark datasets for hallucination detection, standardized validation protocols for multi-modal models).

3. Limited Discussion of Sociotechnical Implementation Factors

While the paper emphasizes technical trust dimensions, factors such as organizational readiness, interoperability, and liability frameworks are underexplored. Incorporating these could make the review more holistic.

4. Clarification on "Stakeholder Trust Requirements" Taxonomy

The distinctions among patient, physician, and regulator trust dimensions are well-articulated but could be summarized more systematically (e.g., in a comparative table or figure). This would make the framework more accessible to readers. Also, from a shareholder perspective to trustworthy AI, is "AI researchers" an shareholder?

5. Quantitative Evidence for Some Claims

Certain statements (e.g., "most AI systems fail to achieve sustained clinical deployment") could be supported with quantitative synthesis or a systematic mapping of clinical adoption rates to strengthen empirical grounding.

6. Citation of Recent Literature

Bridging the gaps: Overcoming challenges of implementing AI in healthcare. Med. 2025
https://pubmed.ncbi.nlm.nih.gov/40220747/
The rise of agentic AI teammates in medicine. The Lancet. 2025
https://pubmed.ncbi.nlm.nih.gov/39922663/

Minor Points

1. Presentation and Readability

Figures could be more visually engaging (e.g., Figure 2 summarizing the framework might use a layered or cyclical design to emphasize iteration). Current scheme suggests that the workflow is linear without feedbacks or iterations, which might be an over-simplification.

Some sections (e.g., 3.2 Uncertainty Quantification) could benefit from schematic diagrams distinguishing epistemic vs. aleatoric uncertainty for clarity.

2. Consistency of Terminology

The terms "trustworthy AI," "trust," and "trustworthiness" are sometimes used interchangeably; a clear working definition early in

the manuscript would improve conceptual precision.

3. Citation Formatting

A few citations appear incomplete (e.g., missing reference number for the MedHalu study citation marked "?"). Ensure all references are verified and consistent with journal style.

4. Style

Some sentences could be shortened for clarity, especially in the introduction and foundation model sections, where long clauses may hinder readability.

The authors may consider moving some technical descriptions (e.g., detailed explanations of SHAP, LIME, TabNet) to a supplementary table summarizing XAI methods.

5. Typo

Minor typographical errors: e.g., "high-dimensionalcontemporary" (missing space, p.7).

Overall Recommendation

This is a well-organized, timely, and insightful review that integrates stakeholder theory with technical and regulatory analyses in healthcare AI. With modest revisions to strengthen empirical grounding for the proposed framework, and improve presentation, this manuscript will make an excellent contribution to the literature on trustworthy and translational medical AI.

Recommendation: Minor to moderate revision.

We thank the reviewers for the constructive feedback. We have addressed all concerns in a point-by-point response below. In particular, we have now added concrete examples and a detailed description of framework operationalization and discussed factors beyond trustworthiness. In addition, we have changed the article type to Perspective.

All changes in the manuscript are highlighted in blue.

Reviewer #1

Comment R1-M1: *Need for a Comprehensive Overview Table. While the text includes numerous illustrative examples, they are dispersed throughout and challenging to track. A summary table consolidating these examples would significantly enhance the reader's understanding of the current landscape of AI in healthcare. The table could include the following columns: clinical task/question, data modality, AI algorithm or architecture, dataset size, validation strategy (internal/external), clinical application context, advantages, and limitations. This will help readers quickly grasp the diversity and maturity of existing applications.*

Reply: We thank the Reviewer for this excellent suggestion. We have added Table 1, which provides a consolidated overview of healthcare AI applications. Given the Perspective format of our article, we prioritized illustrative examples that highlight different trajectories in the research-to-practice gap rather than providing an exhaustive, systematic overview. We adapted the suggested column structure to better align with our focus on trustworthiness and clinical translation: instead of technical details, we included a “Deployment Status” column that directly illustrates whether systems achieved sustained real-world implementation, and a “Key Barrier” column linking each example to the trustworthiness challenges discussed in our manuscript.

Comment R1-M2: *Lack of Concrete, Traceable Case Studies. Several sections, particularly Section 3.1 ("Methodology Overview"), discuss general AI methods such as decision trees and random forests but do not cite specific real-world implementations. Adding traceable examples, including references to published studies or deployed systems, would anchor the discussion and help illustrate the practical challenges these methods face in clinical translation. Consider highlighting a few high-impact use cases (e.g., radiology, pathology, dermatology, or cardiology) where AI has either succeeded or failed in real-world adoption.*

Reply: We thank the Reviewer for this constructive suggestion, which substantially strengthens the empirical grounding of our manuscript. We have made the following additions:

1. Section 3.1 (Explainability): We now include concrete empirical studies evaluating XAI methods with clinician

2. Table 1: Our new overview table provides deployment status and key barriers for representative AI systems across different high-impact use cases (e.g., pathology, dermatology, and oncology)

Comment R1-M3: *Challenge-to-Solution Mapping. Section 5 ("Recommendations for Implementation-Ready Trustworthy AI in Healthcare") offers valuable conceptual guidance, but it lacks direct linkage to the specific barriers identified in Sections 3 and 4. To enhance the paper's practical utility, include a challenge-to-solution mapping table or a straightforward narrative linking each identified challenge (e.g., data heterogeneity, lack of explainability, regulatory uncertainty) to the corresponding recommendation or proposed mitigation strategy.*
Reply:. We have added Table 2, which provides a systematic challenge-to-solution mapping that directly links the implementation barriers identified in Section 3 and 4 to specific recommendations from our five-phse framework in Section 5. For each challenge, we specify which framework phase(s) address it and provide concrete proposed solutions. This explicit mapping demonstrates how our stakeholder-centered framework systematically addresses the implementation barriers documented throughout the manuscript, enhancing the internal coherence of our analysis.

Comment R1-M4: *Figures and Tables Require Strengthening. The visual components are currently underdeveloped. Figures could be made more informative and integrative, summarizing frameworks, data flow, or stakeholder relationships. Tables summarizing XAI methods, regulatory frameworks, or implementation barriers would enhance accessibility and help readers synthesize the dense narrative content.*
Reply: We have now substantially strengthened the visual components of the manuscript: New Tables:

- Table 2: Challenge-to-solution mapping linking implementation barriers from Section 3-4 to specific framework phases, directly addressing the request for implementation barriers synthesis
- Appendix Table 1: Comparison of explainable AI methods in healthcare

Comment R1-M5: *Operationalization of the Proposed Framework. The proposed five-phase stakeholder-centered framework is conceptually well-structured but remains theoretical. The manuscript would benefit from the inclusion of at least one detailed case study or pilot example. For instance, the clinical development of an AI diagnostic tool in radiology or pathology to demonstrate how this framework can be applied in real-world settings, from model design through to regulatory approval and clinical integration.*
Reply: We have added a case study of LumineticsCore (Digital Diagnostics), the first FDA-authorized autonomous AI diagnostic system, which achieved deployment across more than 1,000 U.S. primary care sites. This case study demonstrates how our five-phase framework maps to a successful real-world development trajectory.

Comment R1-M6: *Need for Critical Evaluation of Evidence. Although the manuscript cites extensive secondary literature, much of the discussion remains descriptive. Assertions such as "patients consistently demonstrate willingness to sacrifice accuracy for explainability" should be supported by empirical evidence, ideally surveys or behavioral studies, with commentary on sample diversity, study design, and contextual factors. A more critical appraisal of existing data would substantially increase the review's analytical rigor and credibility.*

Reply: Upon careful examination, we found that our original assertion requires substantial nuance. The empirical evidence is more complex and context-dependent than our original formulation suggested:

1. Supporting evidence (Haggenmüller et al. 2024, JAAD): A prospective multicentric conjoint survey of dermatology patients (n=178) found that patients ranked explainability (26.6%) higher than diagnostic accuracy (20.6%) in relative importance. Furthermore, 41.6% stated they would not rely on AI systems if decisions could not be traced by either clinician or patient. Patients with prior AI experience showed even stronger preferences for explainability (29.5% vs. 16.0% for accuracy). This partially supports our original claim.
2. Contradicting evidence (van der Veer et al. 2021, JAMIA): A UK citizens' jury study (n=36) that forced explicit binary choices found that 86–92% of participants favored accuracy over explainability in life-or-death healthcare scenarios (stroke, kidney transplant). However, in non-healthcare contexts (criminal justice), participants showed opposite preferences.

The apparent contradiction can be explained by: (a) methodological differences (relative importance ratings vs. forced binary choices), (b) different clinical contexts, and (c) stakeholder composition (dermatology patients and physicians vs. general public).

As a consequence, we have substantially revised Section 3.1 to present both sets of findings with appropriate critical appraisal.

Comment R1-M7: *Regulatory Comparison Lacks Depth. The comparison between the FDA and the EU AI Act is insightful but somewhat superficial. The discussion should be expanded to examine how the regulatory philosophies, efficiency-oriented (FDA) versus risk-preventive (EU), affect innovation ecosystems, clinical adoption speed, and patient safety culture. Including a comparative table summarizing these contrasts and their implications for AI development and approval would make this section far more impactful.*

Reply: We have made two changes: First, we expanded the narrative in Section 2.3 to more explicitly contrast how the FDA's efficiency-oritented philosophy versus the EU's risk-preventive approach create different trade-offs for innovation speed, clinical adoption, and patient safety. Seconde, we have added Appendix Table 2, which provides a systematic comparison of both regulatory frameworks across key dimension including approval

pathways, validation requirements, transparency mandates, and implication for AI development and clinical adoption.

Comment R1-M8: *Synthesis of Explainable AI (XAI) Techniques. Section 3.1 lists several explainability techniques (e.g., SHAP, LIME, Grad-CAM) but does not integrate them into a comparative framework. Adding a summary table comparing key XAI methods, including interpretable models such as TabNet, based on criteria such as type (post-hoc vs. intrinsic), interpretability level, computational cost, limitations, and clinical applicability, would provide clarity and practical insight.*
Reply: We have added Appendix Table 1, which provides a comparative overview of explainable AI methods in healthcare applications. The table compares six representative approaches across their mechanisms, strengths, limitations, and available clinical evidence. We did not include TabNet, as our table focuses on explanation methods rather than model architectures.

Comment R1-M9: *Insufficient Discussion on Mitigation of Emergent Behaviors. The section on hallucinations and emergent behaviors of foundation models correctly identifies these as critical risks but lacks discussion of mitigation strategies. The authors should expand this by referencing current best practices such as red-teaming exercises, model auditing, safety benchmarking, and the use of AI system cards for documentation. Positioning the proposed framework in relation to these tools would clarify how it complements or extends existing approaches.*
Reply: We have added a new paragraph in Section 4.1 discussing mitigation strategies for hallucinations and emergent behaviors. This paragraph addresses red-teaming exercises for proactive identification of failure modes, safety benchmarking using dedicated medical hallucination datasets, model auditing for ongoing behavioral assessment, and AI system cards for transparent documentation of model capabilities and limitations.

Comment R1-M10: *Broader Ethical Considerations. While the paper's focus on "trustworthiness" is commendable, it underrepresents related ethical dimensions such as fairness, privacy, accountability, and transparency-all integral to stakeholder trust. These aspects should be explicitly discussed, particularly regarding how regulatory frameworks operationalize them and how they influence clinical acceptance and patient equity.*
Reply: We fully agree that fairness, privacy, accountability, and transparency are integral to trustworthy AI in healthcare. Our manuscript deliberately focuses on three implementation challenges, explainability, uncertainty quantification, and model evaluation. Given the word limit constraints of the Perspective format, which we have already reached, a comprehensive treatment of all ethical dimensions would exceed the feasible scope. However, we have added a brief acknowledgment in Section 3 noting that our analysis focuses on selected technical dimensions while recognizing that broader ethical considerations, including

fairness, privacy, and accountability, represent equally important areas for trustworthy healthcare AI that warrant dedicated examination.

Comment R1-Minor1: *Readability and Sentence Structure. Some sentences are overly long and concept-heavy, affecting readability. For instance, "...with discomfort increasing to 76.6% for 90% accurate unexplainable systems [17]" could be simplified or divided for clarity. A careful editorial pass focusing on sentence flow and paragraph structure would improve accessibility.*
Reply: We have conducted an editorial revision to improve readability, breaking up overly long sentences and simplifying concept-heavy passages. For example, the sentence highlighted by the Reviewer has been divided into two clearer statements.

Comment R1-Minor2: *Clarification of References and Terminology. Certain references are insufficiently contextualized. For example, "Article 13 establishes detailed transparency requirements" should specify that this refers to Article 13 of the EU Artificial Intelligence Act, with a concise summary of its main provisions. Similarly, define abbreviations such as "technical XAI" upon first mention.*
Reply: We have clarified that "Article 13" refers to Article 13 of the EU Artificial Intelligence Act. We have also ensured that the abbreviations are defined upon first use.

Comment R1-Minor3: *Citation Errors. There appears to be a citation error around the MedHalu dataset. Please verify and correct the reference.*
Reply: We thank the Reviewer for identifying this error. We have corrected the citation.

Comment R1-Minor4: *Overly Detailed Methodological Sections. Some methodological explanations, particularly in Section 3, could be shortened or moved to supplementary material to improve the flow and focus of the main text.*
Reply: We have reviewed Section 3 and streamlined several methodological passages to improve readability. However, we retained core methodological descriptions as they provide essential context for understanding the implementation challenges that follow.

Comment R1-Minor5: *Clarify Novel Contributions. The Conclusion should explicitly state how this review differs from prior work on trustworthy AI in healthcare (e.g., through its stakeholder-centered perspective or integration of foundation model analysis).*
Reply: We have clarified the novel contributions of this Perspective in the last paragraph of the introduction.

Comment R1-Minor6: *Figure Citation. Figure 2 is referenced in the captions but omitted in the main text. Please ensure that it is cited and discussed at the appropriate point.*

Reply: We thank the Reviewer for identifying this oversight and have added a citation to Figure 2 in Section 5 where the stakeholder-centered framework is introduced.

Comment R1-Minor7: *Missing Reference. In Section 4.1 ("Medical Hallucination"), the final sentence lacks a reference. Please verify and add the appropriate citation.*
Reply: We thank the Reviewer for identifying this omission and have added the appropriate references to the final sentence of the Medical Hallucination section.

Comment R1-Minor8: *Sentence Length and Readability. Several paragraphs contain lengthy, complex sentences. Breaking them into shorter, more concise statements will improve comprehension and maintain reader engagement.*
Reply: We have conducted a comprehensive editorial revision throughout the manuscript, breaking lengthy sentences into shorter, more concise statements.

Reviewer #2

Comment R2-M1: *Two major aspects are missing: scalability and generalizability.*
Reply: We thank the Reviewer for highlighting these important aspects. Generalizability is addressed in Section 3.3 ("Distribution Shift and Generalization Challenges"), where we discuss performance degradation across heterogeneous clinical environments, mult-site validation approaches, and the systematic mismatch between research validation and regulatory intended use specifications. We agree with the Reviewer that scalability, encompassing technical infrastructure, computational costs, and organizational capacity for large-scale deployment, represents another critical barrier to clinical translation. However, scalability primarily concerns operational and infrastructural dimensions rather than the trustworthiness dimensions that form the focus of this Perspective. We have added a statement in the Conclusion acknowledging that scalability, alongside regulatory compliance and lifecycle management, represents an important area beyond the scope of our analysis that warrants dedicated examination.

Comment R2-M2: *Trustworthiness is a basic requirement for any successful application of health care AI. In the past, this was certainly not always the case, but it is clear as of now. As a consequence, it should not be dismissed anymore. Certainly, if missing, trustworthiness can be one reason for AI not being successfully integrated into real-world applications. Yet, it is by far not the only one and often not the most important reason for lack of implementation and part of the gap. There are important other guardrails for AI to be implemented on the way towards clinical application that are not well addressed in this manuscript about the gap between research projects (often far too small in scope) and real-world applications that have nothing to do with trustworthiness. As written, this review implies that missing trustworthiness explains the gap and fixing solely trustworthiness will overcome this problem.*

Reply: We fully agree with the Reviewer that trustworthiness is a basic requirement for healthcare AI, necessary but not sufficient for successful clinical translation. We did not intend to imply that fixing trustworthiness alone will overcome the research-to-practice gap. We have revised the manuscript to explicitly frame trustworthiness as "a necessary, though not sufficient, condition" (Abstract, Introduction) and added acknowledgment of additional critical factors (including regulatory compliance, lifecycle management, scalability, and data quality control) in the Conclusion. We appreciate the Reviewer's emphasis on this distinction and believe our revisions now better reflect the multifactorial nature of the implementation challenge.

Comment R2-M3: *Other major topics that would have to be addressed, when talking about the gap: 1. The guardrail system of the European AI act has many aspects that are required to close the gap, often seen by academic researchers as imposed hurdles, while in reality most AI modelers just don't understand that AI in medicine cannot be based on "playing around with cool algorithms". 2. The regulations of AI as a medical device. Another area often dismissed by academic AI experts. 3. The lifecycle issue of AI as a health care product, which is another area not well or not at all understood by many academic researchers in the AI model and algorithm space. 4. The lack of understanding of the enormous requirements for data QC in the health care sector, which is dismissed - this reviewer would estimate - by more than 75% of AI modelers in the biomedical AI field.*
Either, the authors choose to address more comprehensively the reasons for the gap or, alternatively, the authors could choose to focus more on the topic trustworthiness and its role in developing AI for medicine and health care. But focusing only on trustworthiness might be seen somewhat as a limitation, as trustworthiness has to be seen as a prerequisite for any future medical AI application and there is quite some literature about this topic already.
Reply: We agree with the factors contributing to the research-to-practice gap. Our manuscript deliberately focuses on trustworthiness as one important dimension. We acknowledge that this focus represents a limitation: addressing trustworthiness alone will not close the gap. We have revised the manuscript to make this scope explicit in the Introduction and to acknowledge these additional factors in the Conclusion. Regarding the existing literature on trustworthiness, our contribution differs through (1) the analysis of stakeholder-specific trust requirements and their misalignment with current research priorities, and (2) the integration of foundation model-specific challenges that have received limited attention in existing literature..

Comment R2-M4: *Paragraph 2.1. While certainly interesting, this topic does only indirectly speak to the gap itself. AI-based medical devices developed as support systems for physician decision making circumvent the issue addressed here already. Mainly due to the fact that experts can cope better with grey box applications, while white box applications are often not performing well. Again, Blackbox applications are not an option for clinical application, if one follows the guardrails as they are outlined*

in e.g., the European AI act or the regulations for AI-based medical devices since explainable AI is a prerequisite for real-world application.

Reply: The reviewer raises an important point that most healthcare AI systems are designed as physician decision support tools where clinicians retain final authority, and that explainable AI is mandated by the EU AI Act.

However, we maintain that Section 2.1 addresses a clinically relevant dimension of the gap since regulatory requirements for explainability differ substantially between jurisdictions, and the assumption that black-box systems are “not an option” does not reflect current US regulatory reality. While the EU AI Act mandates transparency for high-risk AI systems, the FDA takes a markedly different approach. The FDA’s January 2025 draft guidance uses permissive language throughout, stating that transparency and explainability are “important factors” and that FDA “encourages” sponsors to include detailed information. But these remain recommendations, not requirements. Critically, 96.4% of FDA-authorized AI/ML medical devices are cleared via the 510(k) pathway, which requires only “substantial equivalence” to predicate devices without explicit explainability requirements. This regulatory divergence means that black-box systems remain permissible in the US market.

Comment R2-M5: *Paragraph 2.2. Again, while the context is really interesting to read, it does not address the gap directly. In this context, it is also interesting to note that the FDA is aware of the fact that previous authorizations of AI-based medical devices need to be reevaluated since they do not fulfill today's guardrails for AI-based medical support systems anymore. As such, this paragraph addresses mainly the mistakes of the past. The last paragraph of section 2.2 illustrates the reasons for failure. Yet again, this experience has been utilized in updated requirements in the aforementioned guardrails (which are seen as insurmountable obstacles by academic AI experts, mainly because dealing with these issues is not very appealing and reduces the flow towards high-impact publications).*

Reply: The reviewer suggests that Section 2.2 primarily addresses "mistakes of the past" and that the FDA is aware that previous authorizations require reevaluation, with updated guardrails now addressing these issues. We respectfully note that even the updated FDA guardrails remain non-binding recommendations with respect to transparency and explainability. The latest January 2025 draft guidance characterizes these as "important factors" using permissive language ("encourages," "recommends") rather than mandatory requirements. Thus, even for future submissions, explainability remains a recommendation rather than a prerequisite for market authorization, in contrast to the EU AI Act's legally binding transparency requirements. We agree with the reviewer's observation that academic AI researchers often view regulatory requirements as obstacles to high-impact publications. This observation reinforces rather than contradicts our central argument about research-to-

practice misalignment (Section 2.4): current academic incentive structures do not reward the rigorous validation, documentation, and stakeholder engagement required for clinical translation. To address this comment, we have made two revisions to Section 2.3. We have clarified in Section 2.3 that FDA guidance uses permissive language with respect to transparency and explainability rather than mandatory requirements, contrasting with the EU AI Act's legally binding framework. Second, we added a statement emphasizing that the critical challenge is not *whether* transparency and explainability are required, but *how* they are implemented for different stakeholders, a distinction that current regulatory frameworks do not adequately address in our opinion.

Comment R2-M6: *Paragraph 2.3. The last sentence in the section entitles EU AI Act illustrates a little bit the narrative of this review. Of course, this sentence is one explanation, why things are slower, if you have to develop systems that close the gap, while we all actually do not know this yet, since there are no AI-based medical devices put on the market following these new guardrails in Europe. Another way to look at this could be that we need more experts that can address the tasks necessary to elevate an AI-based product from TRL1-3 to TRL7-9. So, the gap is more in how do we really develop the path towards real products. This is less an academic issue, but a translation issue with too few players and too few experts currently available. Other industries are solving similar tasks and they find or even already found solutions.*

Reply: The reviewer correctly notes that no AI-based medical devices have yet been placed on the EU market under the new AI Act guardrails. We acknowledge that our statement about "market access delays" is speculative and should be revised. We appreciate the reviewer's alternative perspective that the gap may primarily reflect insufficient translational capacity, specifically, a shortage of experts capable of advancing AI systems from TRL 1-3 to TRL 7-9, rather than regulatory barriers per se. This framing aligns with our stakeholder-centered framework (Section 5), which emphasizes the need for interdisciplinary teams integrating clinical, regulatory, and technical expertise from early development phases. The reviewer's observation that other industries successfully navigate similar translational challenges suggests that building such capacity is achievable. To address this comment, we have revised the final sentence of the EU AI Act section to remove the speculative claim about market delays and instead emphasize that successful translation will depend on building interdisciplinary translational capacity.

Comment R2-M7:*This reviewer would respectfully disagree with the last sentence in the paragraph "Regulatory Frameworks Versus Physician and Patient Trust Requirements" In my view the European AI Act actually addresses this topic.*

Reply: Upon reflection, we agree that the EU AI Act does address stakeholder-specific transparency requirements, including deployer obligations (Article 13) and affected persons' right to explanation of individual decisions (Article 86). We have removed this paragraph.

Comment R2-M8: *Section 2.4. First Paragraph. This reviewer respectfully disagrees. While there has been an enormous push for autonomous AI products in the past, with the advent of regulations requiring explainable trustworthy AI, this has already shifted towards AI-based products with human oversight AI-based medical support systems. This reviewer would again respectfully disagree with the paragraph entitled "Technical Approval Versus Clinical Acceptance" This paragraph implies to play technical against clinical acceptance values. This is actually not what the guardrails suggest. Both need to be addressed equally. An AI-based test that has low accuracy, even if explainable, will not survive the product development process. However, what seems more important is to address both generalizability and scalability to be included into the development process. Both require that technical and clinical acceptance values are sufficiently high to better estimate real world application. The paragraph entitled "Technical Explainability versus Stakeholder Communication Needs" makes one important statement: "Regulatory frameworks such as the EU AI Act ....". Again, while the provided information is interesting, it distracts from the potential these new guardrails provide to guide AI-development away from the "academic play ground" to real product developments. In this context, it might be more important to mention that purely academically-driven research in this area will likely keep the gap as it is not addressing the next necessary steps, but often fighting them.*

Reply: The reviewer correctly observes that industry has already shifted toward AI-based support systems with human oversight in response to new regulatory requirements, and that our original "versus" framing overstated the tension between dimensions that must be addressed together. We particularly appreciate the reviewer's insight that the gap increasingly reflects a translation issue, with academic research often disconnected from the practical steps required to advance AI systems from early-stage development to market-ready products. This observation aligns with and strengthens our core argument.

To address these points, we have substantially revised Section 2.4:

- Renamed the section to "Misalignment with Current Academic AI Research Priorities" to clarify that our critique targets academic incentive structures, not industry practice
- Replaced "Standalone System Development Versus Human Oversight Requirements" with "Publication-Driven Research Incentives," explicitly addressing how academic incentive structures, including the pressure for rapid, high-impact publications, discourage the time-intensive interdisciplinary collaboration needed for translation
- Renamed "Technical Approval Versus Clinical Acceptance" to "Benchmark Optimization and Real-World Generalizability," removing the adversarial framing and emphasizing that both technical performance and trustworthiness dimensions must be addressed together

Comment R2-M9: *Section 3: The statements in the first sentence of paragraph 3 are debatable. Again, trustworthiness is certainly one major aspect, but not the only one. Focusing*

the discussion in this manuscript only in trustworthiness, with an unexplained introduction of three areas that are addressed (explainability uncertainty quantification, and evaluation) leaves out that other aspects also need to be addressed to close the gap. This reviewer is not convinced that the gap will be closed, even if these points are all addressed.

Reply: We fully agree that trustworthiness is not the only factor contributing to the research-to-practice gap. Other dimensions (including translational capacity, data access, workflow integration, and sustainable business models) are equally important for successful clinical translation. Our manuscript focuses specifically on trustworthiness-related challenges as one contribution to understanding the gap, without claiming that addressing these alone would be sufficient. The selection of explainability, uncertainty quantification, and evaluation follows from the stakeholder requirements identified in Section 2. To clarify this scope limitation, we have revised the manuscript at several points to emphasize that trustworthiness represents a necessary but not sufficient condition for closing the research-to-practice gap. This framing now appears for example in the abstract and we have also revised the opening of Section 3.

Comment R2-M10: *Section 3.3 In the paragraph entitled "Distribution Shift and Generalization Challenges" this reviewer again thinks that the direction is not entirely helpful. The last sentence implies that multi-site validation remains resource-prohibitive, while generalization of AI-based approaches actually requires exactly this. Rather than spending time on synthetic corruption testing, wouldn't it be better to develop the necessary development environment that really address distribution shift and generalization challenges? This reviewer thinks that the manuscript is not sufficiently pro-active into the real-solution aspects. This is similarly true for the paragraph Evaluation of Human-AI-Performance. Yes, the direct comparison is not leading anywhere, but it would be important to better describe what is really required: Physician +/- AI-based support systems, rather than criticizing the past.*

Reply: We thank the reviewer for this constructive feedback, which has helped us make the manuscript more solution-oriented. Regarding synthetic corruption testing: We agree that multi-site validation with diverse real-world data represents the gold standard for assessing generalization. Our intention was not to advocate for synthetic testing as a preferred approach, but to acknowledge it as a pragmatic intermediate step when access to multi-site data is limited, which remains a common constraint in academic settings. We have revised the paragraph to clarify this hierarchy: real-world multi-site validation is the goal, and building the necessary infrastructure should be a field priority; synthetic testing serves as a fallback when data access constraints apply, preferable to no generalization testing at all. Regarding Human-AI Performance evaluation: We fully agree that describing what is required is more valuable than criticizing past approaches. We have rewritten this paragraph to emphasize the "Physician and AI" evaluation paradigm. This framing aligns with the proactive solutions detailed in our framework (Section 5), which emphasizes early clinician involvement and

diverse, representative data throughout development. We believe these revisions better reflect the reviewer's call for a more proactive, solution-oriented perspective.

Comment R2-M11: *In the paragraph "implementation challenges": Again, this reviewer thinks that the authors missed out on opportunities to draw a picture of the future.*
As stated earlier by this reviewer, the real problem is that academic AI modeler are often not interested in bringing their work to biomedicine, but rather cranking papers focusing on one algorithm after the other on medically often meaningless - often open access - datasets. This focus on paper rather than impact creation is one main reason for the gap. As long as the sentence "I don't want to deal with physicians, I just need some data to try my best algorithm" is a standard in the field of academic AI research, the gap will not close.
If at all, one needs to sketch roadmaps, how those experts that handle the medical tasks (first of all physicians) enter into meaningful discussions with data scientists to generate AI-ready data, followed by data analysts to provide QC-ed data to model experts that develop AI based on best model + best data. This only is provided in section 5 without a lot of detail of the "how to". We need standing networks across institutions that address these issues in secured environments where experts can work together despite existing regulations that might be experienced as hurdles, instead of trying to address them in single center AI-studies.
Reply: This feedback resonates strongly with our analysis. Regarding the academic incentive problem: We fully agree that the attitude "I don't want to deal with physicians, I just need some data" represents a fundamental barrier to translation. We have now explicitly addressed this in our revised Section 2.4 ("Publication-Driven Research Incentives"), which describes how academic incentive structures favor rapid publication over the time-intensive process of building interdisciplinary teams. Regarding the need for concrete roadmaps and institutional infrastructure: We agree that Section 5 required more detail on the "how to." We have expanded Phase 1 ("Forming Interdisciplinary Teams") to address the institutional infrastructure needed for sustained collaboration. The revised text now discusses standing networks across institutions, secure data environments enabling multi-site collaboration, and the role of funding agencies in incentivizing translation-oriented research over single-center algorithm development.

Comment R2-M12: *Paragraph 4: The first paragraph under 4.1. nicely summarizes the enormous difference between foundation models and conventional AI. This reviewer would strongly suggest to focus on conventional AI. The well-made points of this short two sentences question trustworthiness in foundation models significantly. Sometimes less is more.*
Reply: We respectfully prefer to retain the foundation model section for the following reasons: Foundation models are currently receiving enormous attention in both AI research and healthcare applications, being actively explored for clinical deployment. A perspective on trustworthy AI in healthcare that omits this rapidly evolving area would risk being incomplete.

Comment R2-M13: *Paragraph 5: While this reviewer actually likes the idea to provide a framework for development, the proposed structure misses out on important aspects.*
** Link to TRL system (???)*
** Link to regulatory frameworks and guardrails*
** Link to development paths for products (AI-based medical support systems)*
** Aspects of product life cycle, different kinds of shifts (distribution shifts, etc.)*
Reply: Our primary target audience is academic AI researchers who wish to design their studies from the outset in a way that enables subsequent translation and product development. For this audience, the framework highlights trustworthiness considerations that are often overlooked in technically-focused academic research but are essential prerequisites for later regulatory approval and clinical deployment. A full framework encompassing TRL mapping, regulatory checkpoints, commercialization pathways, and lifecycle management would constitute a substantial undertaking beyond the scope of this perspective. Our contribution is to identify where trustworthiness-specific considerations must be embedded early in the research process, so that academic outputs can serve as a foundation for subsequent product development rather than remaining isolated research artifacts. We have added a brief statement in Section 5 clarifying the intended audience and scope of the framework.

Comment R2-M14: *While the text for the five phases is easy to understand, it stays often rather vague. E.g., for validation, there are already clearly defined regulations and pathways towards product development that need to be fulfilled and this would need some more description of the components. There is no doubt that phase 5 validation is of utmost importance, but it remains unclear from the text in this manuscript how this would look like in a project that would close the gap.*
Reply: We acknowledge that Phase 5 (Validation) remains at a relatively high level of abstraction. As noted in our response to the previous comment, this framework targets academic AI researchers who wish to design translation-ready studies, rather than providing a comprehensive product development guide. Detailed validation protocols, regulatory pathways, and certification requirements are extensively documented in regulatory guidance and would exceed the scope of this review.

Reviewer #3

Comment R3-M1: *Framework Validation. The proposed five-phase framework is conceptually sound, but it remains theoretical. The authors could strengthen their argument by referencing any empirical or case-study evidence where similar stakeholder-driven frameworks have*

improved AI deployment (e.g., clinical AI co-design studies). Furthermore, possible feedbacks/feedforwards/iterations of the proposed five phases should be discussed.
Reply: We thank the reviewer for these constructive suggestions. Regarding empirical evidence: The revised manuscript now includes a case study of LunimeticsCore, the first FDA-authorized autonomous AI diagnostic system. Regarding iterations and feedback loops: We agree that the linear presentation of the five phases may obscure the iterative nature of real-world development. However, we would emphasize that not all iterations are desirable. A common anti-pattern in academic research is adapting the problem definition to available data. For example, reformulating the clinical question to fit a convenience dataset rather than collecting data appropriate to a well-defined clinical need. Our framework explicitly discourages such iterations: the intended use specification (Phase 2) should be defined collaboratively with clinical experts before data collection and should remain stable thereafter. Legitimate iterations, by contrast, occur when later phases reveal genuine issues, for example, usability testing (Phase 4) uncovering workflow integration problems, or validation (Phase 5) identifying performance gaps in specific subgroups that necessitate additional data collection (Phase 3). We have added a paragraph in Section 5 clarifying this distinction between appropriate feedback loops and problematic post-hoc adjustments.

Comment R3-M2: *Depth of Discussion on Foundation Models. The section on foundation models identifies key risks (hallucinations, emergent behaviors, data leakage) but could be strengthened by suggesting practical mitigation strategies (e.g., benchmark datasets for hallucination detection, standardized validation protocols for multi-modal models).*
Reply: We thank the reviewer for this suggestion. We have added a new subsection "Mitigation Strategies" in Section 4 that addresses practical approaches to foundation model risks. This includes red-teaming exercises for proactive identification of failure modes, safety benchmarking using dedicated medical hallucination datasets (e.g., MedHaLu), model auditing for ongoing assessment, and transparency documentation through model cards to communicate capabilities and limitations to clinical end-users. We also emphasize that even comprehensive mitigation strategies cannot guarantee the absence of novel failure modes given the fundamental unpredictability of emergent behaviors, underscoring the continued importance of human oversight.

Comment R3-M3: *Limited Discussion of Sociotechnical Implementation Factors. While the paper emphasizes technical trust dimensions, factors such as organizational readiness, interoperability, and liability frameworks are underexplored. Incorporating these could make the review more holistic.*
Reply: We thank the reviewer for highlighting these important dimensions. We agree that organizational readiness, interoperability, and liability frameworks represent critical factors for successful clinical AI implementation. However, our framework is designed for academic AI researchers who wish to design their studies from the outset in a way that enables subsequent translation. Sociotechnical implementation factors such as organizational change

management, IT infrastructure integration, and legal liability allocation, while essential for deployment, typically fall outside the direct influence of academic researchers. We have added a statement in Section 5 clarifying that this framework specifically targets academic research, enabling researchers to design studies that are translational-ready from the outset, so that subsequent product development can build upon rather than discard academic outputs.

Comment R3-M4: *Clarification on "Stakeholder Trust Requirements" Taxonomy. The distinctions among patient, physician, and regulator trust dimensions are well-articulated but could be summarized more systematically (e.g., in a comparative table or figure). This would make the framework more accessible to readers. Also, from a shareholder perspective to trustworthy AI, is "AI researchers" an shareholder?*
Reply: We thank the reviewer for these suggestions. Regarding a systematic summary of stakeholder trust requirements: We appreciate the suggestion for a comparative table. However, as a Perspective article with limited space, we have aimed to keep Section 2 concise and have actually condensed rather than expanded this section during revision. We believe the current narrative structure adequately conveys the key distinctions between patient, physician, and regulator trust dimensions for our purposes. Regarding AI researchers as stakeholders: This is an insightful question. In our framework, we distinguish between stakeholders of trustworthy AI systems (patients, physicians, regulators—those who use, are affected by, or oversee these systems) and the target audience for our framework (academic AI researchers). Researchers are not stakeholders in the traditional sense of having trust requirements that AI systems must satisfy; rather, they are the developers whose work must address stakeholder requirements.

Comment R3-M5: *Quantitative Evidence for Some Claims. Certain statements (e.g., "most AI systems fail to achieve sustained clinical deployment") could be supported with quantitative synthesis or a systematic mapping of clinical adoption rates to strengthen empirical grounding.*
Reply: We thank the reviewer for this suggestion. We agree that quantitative evidence strengthens empirical grounding. The revised manuscript now provides quantitative support for key claims, including the new Table 1 with specific deployment outcomes and validation practices.

Comment R3-M6: *Citation of Recent Literature.Bridging the gaps: Overcoming challenges of implementing AI in healthcare. Med. 2025 https://pubmed.ncbi.nlm.nih.gov/40220747/;*
The rise of agentic AI teammates in medicine. The Lancet. 2025
https://pubmed.ncbi.nlm.nih.gov/39922663/
Reply: We have incorporated both references into the manuscript.

Comment R3-Minor1: *Presentation and Readability. Figures could be more visually engaging (e.g., Figure 2 summarizing the framework might use a layered or cyclical design to emphasize iteration). Current scheme suggests that the workflow is linear without feedbacks or iterations, which might be an over-simplification. Some sections (e.g., 3.2 Uncertainty Quantification) could benefit from schematic diagrams distinguishing epistemic vs. aleatoric uncertainty for clarity.*

Reply: We thank the reviewer for these suggestions to improve visual presentation. Regarding Figure 2 and the linear appearance of the framework: We agree that the original figure may have understated the iterative nature of the development process. We have added a new subsection "Iterations and Feedback Loops" in Section 5 that explicitly addresses this point, distinguishing between appropriate iterations (e.g., usability testing revealing workflow issues) and inappropriate iterations (e.g., adapting the problem definition to fit available data). Regarding schematic diagrams for epistemic vs. aleatoric uncertainty in Section 3.2: We appreciate this suggestion. However, as a Perspective article with space constraints, we have opted to clarify this distinction textually rather than adding additional figures. We have revised the relevant passage to more clearly delineate the two uncertainty types and their differing clinical implications.

Comment R3-Minor2: *Consistency of Terminology. The terms "trustworthy AI," "trust," and "trustworthiness" are sometimes used interchangeably; a clear working definition early in the manuscript would improve conceptual precision.*

Reply: We thank the reviewer for this important point regarding consistency of terminology. We have added a clarifying statement in the Introduction that distinguishes between these related concepts.

Comment R3-Minor3: *Citation Formatting. A few citations appear incomplete (e.g., missing reference number for the MedHalu study citation marked "?"). Ensure all references are verified and consistent with journal style.*

Reply: We thank the reviewer for catching this error. We have corrected the incomplete citation for the MedHalu study and verified that all references are complete and consistent.

Comment R3-Minor4: *Style. Some sentences could be shortened for clarity, especially in the introduction and foundation model sections, where long clauses may hinder readability. The authors may consider moving some technical descriptions (e.g., detailed explanations of SHAP, LIME, TabNet) to a supplementary table summarizing XAI methods.*

Reply: We thank the reviewer for these suggestions regarding readability. We have revised the Introduction and Foundation Model sections to shorten overly long sentences and improve clarity. Regarding the suggestion to move technical descriptions of XAI methods (SHAP, LIME, TabNet) to a supplementary table: As a Perspective article with limited space, we have aimed to keep the methodological overview concise while providing sufficient

context for readers unfamiliar with these methods. We have condensed the technical descriptions in Section 3.1 to improve readability while retaining the essential information in the main text.

Comment R3-Minor5: *Typo. Minor typographical errors: e.g., "high-dimensionalcontemporary" (missing space, p.7).*

Reply: We thank the reviewer for the careful reading. We have corrected this typographical error.

5th Apr 2026

Manuscript Number: MSB-2025-13347R
Title: Toward Trustworthy Healthcare AI: Designing Academic Research for Translation Readiness
Author: Achim Hekler
Florian Buettner

Dear Florian,

Thank you for submitting the revised version of your manuscript. First of all, I apologize for the slow process, which was due to the late arrival of reviewer reports. We have received feedback from two of the three reviewers who agreed to evaluate your revision. Despite several reminders, we were unable to obtain a report from Reviewer #2. Since the other two reviewers provided similar feedback, we have decided to proceed based on their reports. As you will see from their comments below, both are supportive of publication. Before we can formally accept your manuscript, please address the remaining minor issue raised by Reviewer #3, as well as the following editorial-level concerns:

1. If possible, please submit the manuscript in .docx format. Please remove all figures from the main manuscript file.

2.Please remove "Appendix A" from the manuscript. This table should be renamed "Table EV1" and uploaded as a separate file(.xls format). The file should include an additional sheet labeled "Legend" containing the corresponding table legend. Please also update the in-text callout to "Table EV1."

3. Add a missing callout for Table 2.

4. The references need to be formatted according to the Molecular Systems Biology reference style. Citations should be listed in alphabetical order.

5. Grant 101088594 is mentioned in the Acknowledgements but has not been entered in the submission system. Please add it there.

6. section order: Disclosure and Competing Interests Statement should follow the Acknowledgments. Figure legends should come after the References, with tables placed at the very end.

I have forwarded your figures to our designer, who will reach out to you directly for final approval once the figures have been redrawn.

As a matter of course, please make sure that you have correctly followed the instructions for authors as given on the submission website.

Thank you for writing this paper to Molecular Systems Biology.

Kind regards,
Jingyi

Jingyi Hou, PhD
Senior Editor
Molecular Systems Biology

If you do choose to resubmit, please click on the link below to submit the revision online before 5th May 2026.

*** PLEASE NOTE *** As part of the EMBO Press transparent editorial process initiative (see our Editorial at https://dx.doi.org/10.1038/msb.2010.72 , Molecular Systems Biology will publish online a Review Process File to accompany accepted manuscripts. When preparing your letter of response, please be aware that in the event of acceptance, your cover letter/point-by-point document will be included as part of this File, which will be available to the scientific community. More information about this initiative is available in our Instructions to Authors. If you have any questions about this initiative, please contact the editorial office (msb@embo.org).

Reviewer #1:

I recommend the publication of this article in its current form. Such a timely review paper.

Reviewer #3:

The authors changed the article type to Perspective and have addressed the major concerns raised in previous review. The manuscript has been substantially improved and aligned well with Perspective. The authors present a well-structured and well-argued framework and pathway to guide academic AI research design, taking trust requirements into considering at early stage and aiming for future translation readiness. The addition of real-world case and tables improved illustration of viewpoints. I recommend accept with minor revisions.

Typos:
1. Page 6, tFDA Pathway
2. Page 7, his comprehensive approach better aligns. Missing "T"
3. Page 11, line 4: CAI?
4. Page 23, while ... this five-phase approach, missing comma

Follow up email from Editor **18th Apr 2026**

Dear Florian,

I just wanted to let you know that Reviewer #2 has now submitted their report. I have included it below for your reference.

Looking forward to receiving your revised manuscript soon.

Best wishes,
Jingyi

Jingyi Hou
Senior Editor
Molecular Systems Biology

Reviewer #2

(Report for Author)
The authors have made considerable changes based on all three reviewers comments and to this reviewer's opinion, the manuscript has significantly improved.

There are some typos in the newly added text, which require further attention. Other than that, this reviewer has no more further comments for improving the manuscript.

All minor editorial requests have been addressed by the authors.

MSB-2025-13347RR
24th Apr 2026

Dear Florian,

Thank you again for submitting your revised manuscript. I am pleased to inform you that your paper has been accepted for publication.

Should you be planning a Press Release on your article, please get in contact with embo_production@springernature.com after the manuscript is exported, in order to coordinate publication and release dates.

Thank you for your valuable contribution to Molecular Systems Biology!

Kind regards,
Jingyi

Jingyi Hou, PhD
Senior Editor
Molecular Systems Biology
